# Antioxidant and Antimicrobial Activities of Essential Oil Extracted from Leaves of African Boxthorn (*Lycium ferocissimum*): In-Vitro and In-Silico Approaches

**DOI:** 10.3390/molecules31183224

**Published:** 2026-09-13

**Authors:** Walid El Mjiyad, Hind Zejli, Meriem Khedraoui, Outmane El Omry, Abderrahim El Yahyaoui, Khalid Chebbac, Mourad Akdad, Salaheddine Chebaibi, Mourad A. M. Aboul-Soud, John P. Giesy, Mohammed Chalkha, Dunia A. Al Farraj, Samir Chtita, Mohammed L’bachir El Kbiach, Brahim El Bouzdoudi, Abdelfattah El Moussaoui

**Affiliations:** 1 Plant Biotechnology Team, Faculty of Sciences, Abdelmalek Essaadi University, Tetouan 93002, Morocco; walid.elmjiyad@etu.uae.ac.ma (W.E.M.); salaheddine.chebaibi@etu.uae.ac.ma (S.C.); melkbiach@uae.ac.ma (M.L.E.K.); belbouzdoudi@uae.ac.ma (B.E.B.); 2 Laboratory of Engineering, Electrochemistry, Modeling and Environment, Faculty of Sciences Dhar El Mahraz, Sidi Mohammed Ben Abdallah University, P.O. Box 1796 (Atlas), Fez 30000, Morocco; hind.zejli@usmba.ac.ma; 3 Laboratory of Analytical and Molecular Chemistry, Faculty of Sciences Ben M’Sik, Hassan II University of Casablanca, Casablanca 20360, Morocco; meriemkhedraoui5@gmail.com (M.K.); samirchtita@gmail.com (S.C.); 4 Laboratory of Biotechnology and Environment, Agri-Food and Health, Faculty of Sciences Dhar El Mahraz, Sidi Mohammed Ben Abdellah University, P.O. Box 1796 (Atlas), Fez 30000, Morocco; outmane.elomry@usmba.ac.ma; 5 Ecology Unit, Department of Plant Protection and Environment, National School of Agriculture of Meknes, BP S 40, Meknes 50001, Morocco; 6 Laboratory of Biotechnology, Conservation and Valorisation of Natural Resources, Faculty of Sciences Dhar El Mahraz, Sidi Mohammed Ben Abdallah University, Fez 30000, Morocco; abderrahim.elyahyaoui@usmba.ac.ma (A.E.Y.); khalid.chebbac@usmba.ac.ma (K.C.); 7 Department of Biology, Faculty of Sciences and Techniques Errachidia, Moulay Ismail University of Meknes, BP 509, Boutalamine, Errachidia 52000, Morocco; akdad.mourad@gmail.com; 8 Center of Excellence in Biotechnology Research, College of Applied Medical Sciences, King Saud University, Riyadh 12372, Saudi Arabia; maboulsoud@ksu.edu.sa; 9 School of Earth and Environmental Sciences, Baylor University, Waco, TX 76798, USA; jgiesy@aol.com; 10 Department of Veterinary Biomedical Sciences and Toxicology Centre, University of Saskatchewan, Saskatoon, SK S7N 5B3, Canada; 11 Department of Integrative Biology and Center for Integrative Toxicology, Michigan State University, East Lansing, MI 48823, USA; 12 Laboratory of Materials Engineering for the Environment and Natural Resources, Faculty of Sciences and Techniques, University of Moulay Ismail of Meknes, B.P 509, Boutalamine, Errachidia 52000, Morocco; 13 Department of Botany and Microbiology, College of Science, King Saud University, P.O. Box 2455, Riyadh 11451, Saudi Arabia; dfarraj@ksu.edu.sa

**Keywords:** *Lycium ferocissimum*, essential oil, antioxidant and antimicrobial properties, in silico studies

## Abstract

The African boxthorn (*Lycium ferocissimum*) is a medicinal plant belonging to the Solanaceae family that is used in traditional pharmacopeia. Here, its biological potential was assessed by analyzing the essential oil from its leaves (EOLF), extracted by hydro-distillation and major terpenes characterized by gas chromatography coupled to mass spectrometry (GC-MS). GC-MS analysis of EOLF identified fourteen compounds representing 99.95% of the total mass, which was dominated by carvacrol (35.96%) and sabinene (17.06%). EOLF exhibited antioxidant activity as characterized by several assays, including 2,2-diphenyl-1-picrylhydrazyl (DPPH), Ferric Reducing Antioxidant Power (FRAP), Total Antioxidant Capacity (TAC) and the *β*-carotene/linoleic acid test. According to the results obtained, EOLF exhibited antioxidant activity, as measured by the DPPH assay, with a mean IC_50_ of 96 µg/mL, which is relatively close to that of quercetin (74 µg/mL) and BHT (49.16 µg/mL). Furthermore, the activity assessed using the FRAP method showed an EC_50_ of 282 µg/mL, comparable to that of BHT (292 µg/mL). Based on the results of the TAC method, the antioxidant activity was 880 ± 4 µg AAE/mg, while the efficacy for inhibition of β-carotene was 80%. Effective antimicrobial activity against several bacterial and fungal strains was assessed by measuring inhibition zones and Minimum Inhibitory Concentrations (MICs). EOLF exhibited significant antimicrobial activity, particularly against *Klebsiella pneumoniae*, with an inhibition zone of 25.08 ± 0.93 mm and an MIC of 15.68 ± 0.83 µg/mL. For *Staphylococcus aureus*, an inhibition zone of 23.84 ± 0.88 mm and an MIC of 11.03 ± 0.27 µg/mL were recorded. Strong antifungal activity was also observed against *Candida albicans*, with an inhibition zone of 22.55 ± 0.87 mm and an MIC of 10.51 ± 0.47 µg/mL. The conducted in-silico analysis confirmed the affinity of the main compounds with key biological targets, suggesting their likely roles in the observed antimicrobial effects. The ADEM-Tox properties demonstrated that the analyzed compounds would only be lethal at very great concentrations, indicating low acute toxicity, and would not be expected to elicit toxic effects at therapeutic doses.

## 1. Introduction

The growing incidence of adverse effects associated with conventional pharmacotherapies, together with the alarming rise in antimicrobial resistance, represents a major global health challenge. Drug-related toxicity remains a significant cause of treatment discontinuation and morbidity [1], while the rapid evolution of multidrug-resistant pathogens continues to undermine the efficacy of existing antibiotics [2]. These issues highlight the urgent need for new therapeutic agents that are both safer and more effective. In response to this challenge, alternative therapeutic approaches based on natural resources, particularly medicinal plants, have gained considerable attention. Extensive research efforts have been directed toward developing new drugs or therapeutic strategies and identifying effective alternatives to currently used medications. Moreover, herbal remedies have been employed for centuries in the treatment and management of a wide range of diseases.

The genus *Lycium* comprises roughly 90 species with a broad geographic distribution and centers of diversity located in southern South America, southwestern North America, and southern Africa [3,4]. Pharmacological studies have demonstrated that *Lycium* species possess various biological activities, including anti-inflammatory effects [5,6], and modulation of gut microbiota [7]. Phytochemical investigations of *Lycium* species have shown that they are rich in polysaccharides, lipids, terpenes, and phenolic compounds. A wide range of specialized metabolites has been identified in this genus, including glycerogalactolipids, phenylpropanoids, coumarins, lignans, flavonoids, amides, alkaloids, anthraquinones, organic acids, terpenoids, steroids, and their derivatives [8,9].

The African boxthorn (*Lycium ferocissimum*) is a spiny shrub in the nightshade family (Solanaceae) native to South Africa that has been widely introduced across Africa and is used as a medicinal plant [3]. This plant remains poorly studied, and to date only one investigation has described its chemical composition and the antioxidant, cytotoxic, and anti-inflammatory activities of ethanol and aqueous extracts derived from its raw fruits, ripe fruits, and leaves [10]. To the best of our knowledge, this is the first report on the composition and medicinal properties of the essential oil of leaves of *L. ferocissimum* (EOLF) and focused on the extraction and characterization of the chemical composition of EOLF, by use of GC-MS. In vitro antioxidant activity was evaluated by several assays, including DPPH, FRAP, TAC and the *β*-carotene/linoleic acid test. Antimicrobial potential was studied in vitro using the agar diffusion method to determine its antimicrobial properties, as well as in silico through molecular docking and molecular dynamics simulations, and ADMET predictions.

## 2. Results and Discussion

### 2.1. Phytochemical Screening

**Figure 1:**
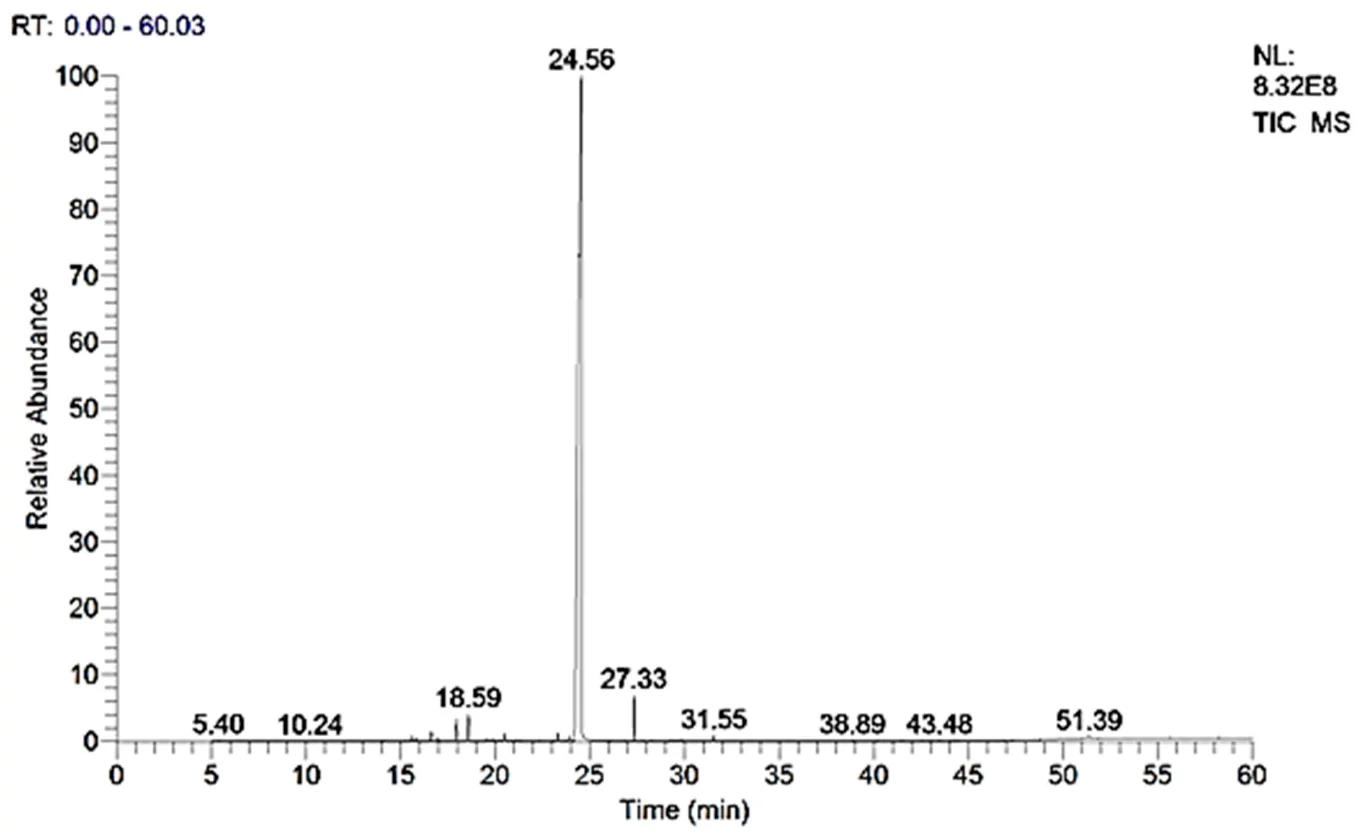
GC-MS profile of EOLF.

The extraction yield for EOLFs was in the region of 0.18%. This Eo contained 14 primary compounds (Figure 1), which accounted for 99.954% of the mass of constituents in EOLF. The chemical composition reveals a predominance of phenolic and terpenic compounds, suggesting significant bioactive potential, particularly in terms of antioxidant, antimicrobial and aromatic activity, justifying interest in pharmacological and industrial applications. The main constituents and their chemical formulas, retention times and relative abundance are summarised (Table 1). Carvacrol or cymophenol (C6H3C3H7; CAS: 499-75-2) was the most abundant compound with a relative abundance of 35.963% and likely accounted for the aromatic and biological properties of EOLF. Other significant compounds included cis-sabinene (17.063%), caryophyllene (15.376%), and its oxidised derivative, caryophyllene oxide (10.921%), as well as α-campholenol (6.157%), isoborneol (4.983%) and borneol (2.897%). Other compounds present in smaller proportions include p-cymene (1.364%), ᴕ-terpinene (1.423%), myrtenyl acetate (1.684%), γ-mamliene (1.637%), eucalyptol (0.125%), isopulegone (0.143%) and valencene (0.218%).

### 2.2. Anti-Radical Activities of EOLF

**Figure 2:**
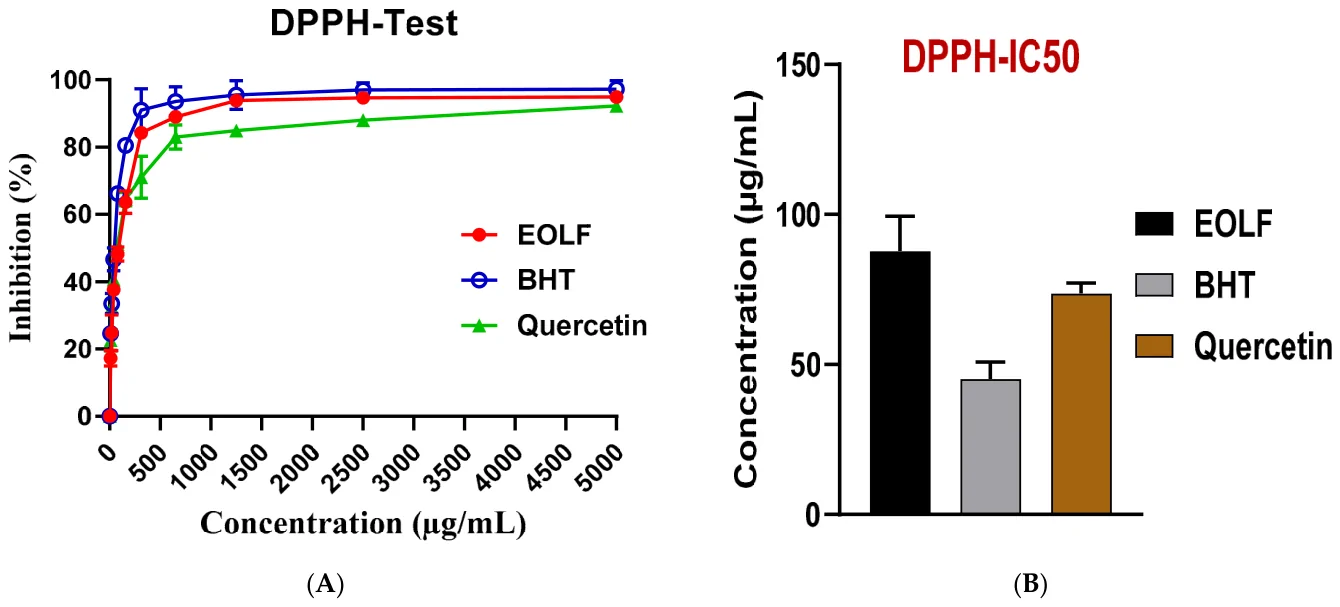
Antioxidant capacity of EOLF, BHT and quercetin evaluated by the DPPH method: (**A**) dose dependence and (**B**) IC_50_.

The antioxidant activity of EOLF, evaluated by the DPPH test, was proportional to concentration, and at 10 µg EOLF/mL, the inhibition of the DPPH radical was moderate (17.27 ± 2.38%), slightly less than that observed for BHT (24.58 ± 0.39%) or quercetin (22.58 ± 3.23%), which indicated that the antioxidant effect of EOLF was detectable, but limited, at this dose. At 312.5 µg EOLF/mL, inhibition increased significantly (84.24 ± 0.85% for EOLF), approaching BHT (90.97 ± 6.45%) and exceeding quercetin (70.97 ± 6.47%), suggesting that the active compounds in the oil, particularly phenols and terpenes, exert an effective antioxidant action at this concentration. At great concentrations (5000 µg/mL), all samples reach a great inhibition plateau, with EOLF at 94.85 ± 0.85%, BHT at 97.21 ± 2.55% and quercetin at 92.21 ± 1.72%, indicating that the maximum effect on free radical neutralization was achieved (Figure 2).

EOLF exhibited significant antioxidant activity, with an average IC_50_ of approximately 96 µg/mL (0.096 mg/mL), which is significantly less than those reported for extracts of *L. ferocissimum* previously. The IC_50_ values for extracts of *L. ferocissimum* have been reported to range from 0.57 to 8.003 mg/mL [10] and 0.65 to 2.15 mg/mL for DPPH radical scavenging [11]. Comparatively, the IC_50_ obtained in the study, the results of which are reported here, was approximately 6- to 80-fold less. The antioxidant capacity of *L. barbarum* fruit depends on concentration, with inhibition percentages of 70.58%, 65.21%, 59.94% and 52.99% at high concentrations of 10, 20, 40 and 50 mg/mL, respectively [12], which is consistent with moderate activity compared to that observed for EOLF at lesser concentrations.

**Figure 3:**
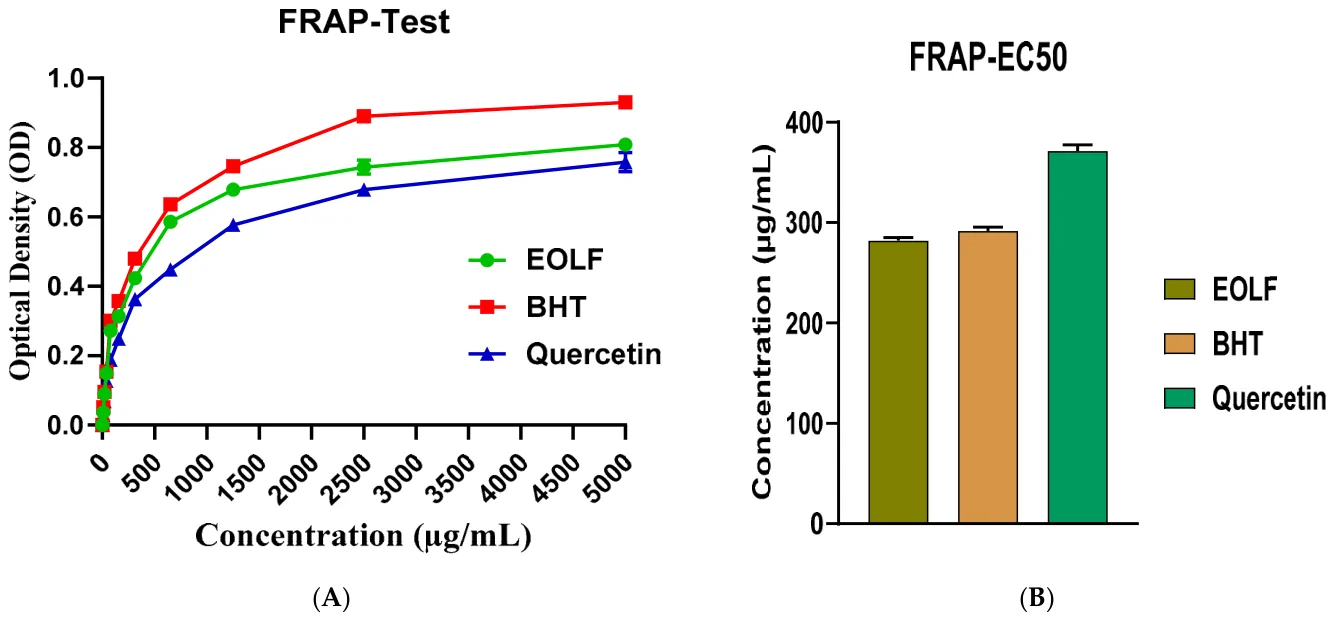
Reducing power of EOLF, BHT and quercetin evaluated by the FRAP method: (**A**) dose dependence and (**B**) EC_50._

The antioxidant activity of EOLF evaluated using the FRAP method shows a dose-dependent increase in reducing capacity (Figure 3A). At 10 µg EOLF/mL, antioxidant activity was low, with a mean optical density (OD) of 0.036 ± 0.002, which is slightly less than that of BHT (0.052 ± 0.005), but comparable to quercetin (0.029 ± 0.001). At medium concentrations (312.5 µg EOLF/mL), the absorbance was 0.424 ± 0.007, remaining slightly less than BHT (0.480 ± 0.006) but greater than quercetin (0.363 ± 0.004), which indicated moderate to relatively great antioxidant activity. At 5000 µg/mL, all compounds reached a plateau, with EOLF (0.809 ± 0.006) similar to the maximum efficacy of quercetin (0.738 ± 0.009) but slightly less than that of BHT (0.930 ± 0.000), which confirmed that maximum efficacy could be achieved at greater doses (Figure 3). The EC_50_ values confirm these observations: EOLF has an average EC_50_ of approximately 282 µg/mL (Figure 3B), indicating significant antioxidant activity but slightly lower than that of the BHT standards (292 µg/mL) and greater than quercetin (371 µg/mL). This activity is likely related to the presence of phenolic and terpenic compounds in the essential oil, such as carvacrol and cis-sabinene. The dose-dependent response and the plateau observed at greater concentrations reflect EOLF’s ability to effectively neutralize free radicals by reducing iron (III) to iron (II).

Although the essential oil from *L. ferocissimum* leaves also exhibits notable antioxidant activity, it remains slightly inferior to that of *L. ruthenicum*, probably due to a lower content of phenolic compounds and other reduced molecules. These observations demonstrate the decisive role of the specific chemical composition of *Lycium* species in their antioxidant power [13].

The evaluation of the total antioxidant capacity (TAC) of EOLF using the phosphomolybdate method shows a dose-dependent activity. At 100 µg EOLF/mL, moderate activity with average values of 108 ± 9 µg AAE/mg was observed, which was slightly less than BHT (125 ± 6 µg AAE/mg) and comparable to quercetin (119 ± 7 µg AAE/mg).

**Figure 4:**
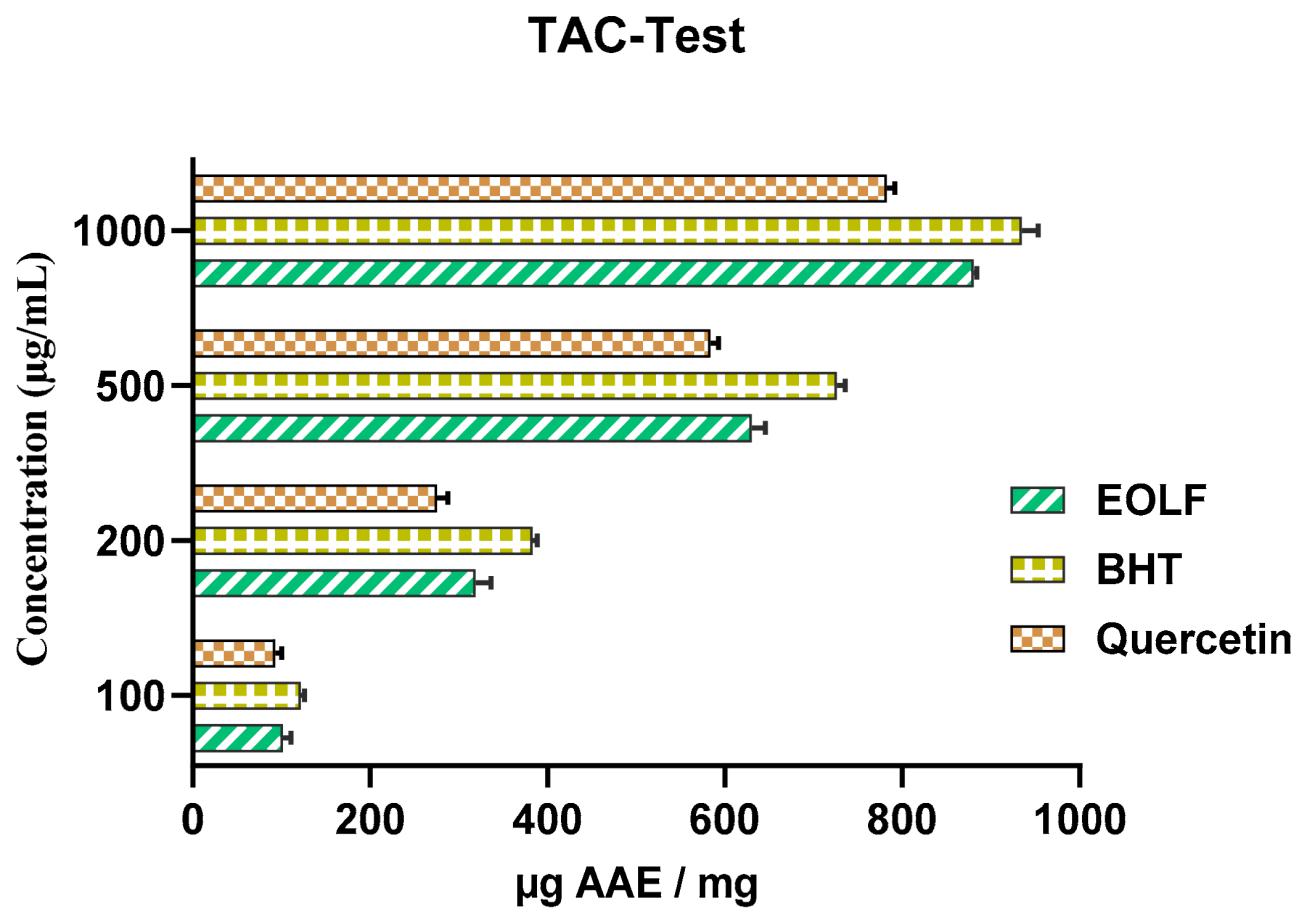
Antioxidant activity of EOLF assessed by the phosphomolybdate method (TAC).

At intermediate concentrations (200 µg/mL), activity was significantly greater, with EOLF reaching 319 ± 17 µg AAE/mg, close to quercetin (276 ± 12 µg AAE/mg) but slightly less than that of BHT (383 ± 6 µg AAE/mg). At 500 µg EOLF/mL, significant antioxidant capacity (630 ± 11 µg AAE/mg) was observed, but this was still less than that of BHT (727 ± 7 µg AAE/mg), and greater than that of quercetin (584 ± 7 µg AAE/mg) (Figure 4). At 1000 µg EOLF/mL, a plateau with a maximum activity of 880 ± 4 µg AAE/mg was observed, which indicates antioxidant potential close to that of the standards. Thus, it can be concluded that EOLF has a total antioxidant capacity comparable to standard synthetic and natural compounds. The dose-dependent progression reflects the cumulative effect of the active compounds present in the essential oil, particularly phenols and terpenes, such as carvacrol and cis-sabinene.

**Figure 5:**
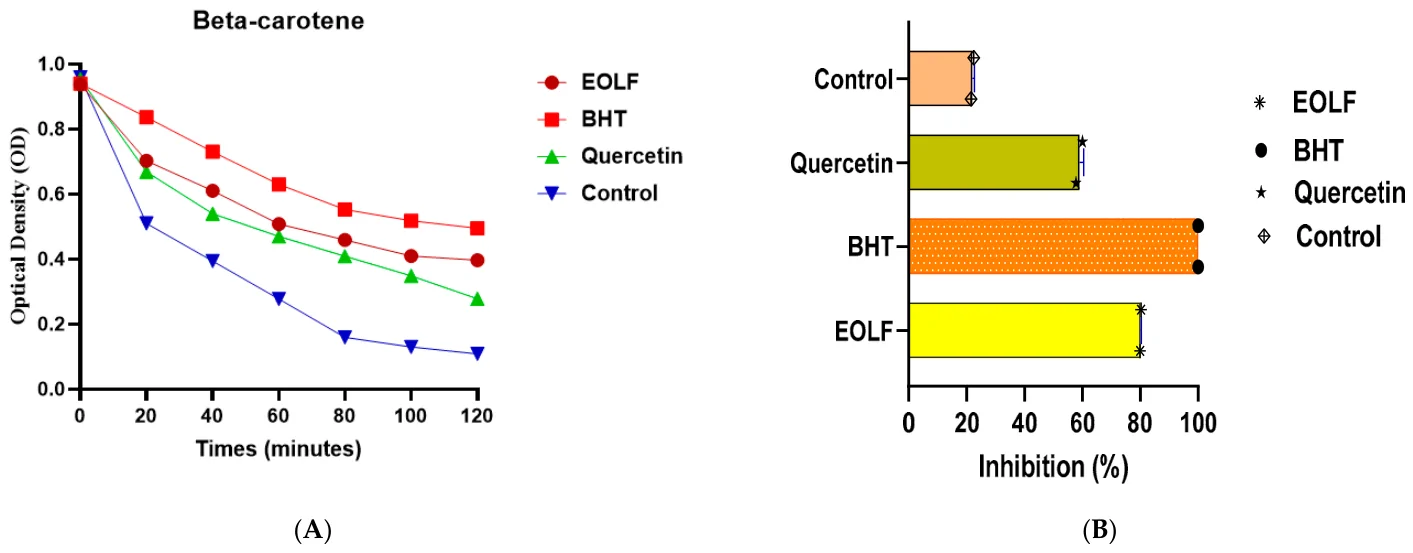
Absorbance of β-carotene at 470 nm in the presence of EOLF, BHT, quercetin and a negative control: (**A**) dose dependence and (**B**) percentage inhibition effect.

When the antioxidant scavenging potential of EOLF was evaluated using the β-carotene discoloration test, which measures the ability to inhibit lipid oxidation over time (minutes), there was a gradual decrease in *β*-carotene absorbance, reflecting dose- and time-dependent protection against oxidation. At shorter intervals, such as 20 min, EOLF exhibited moderate inhibition with a mean absorbance of 0.703 ± 0.002, which was slightly less than that of BHT (0.837 ± 0.007), but greater than the untreated control (0.51 ± 0.01) and slightly greater than quercetin (0.669 ± 0.006) (Figure 5). At an intermediate duration of 60 min, the absorbance decreases for EOLF (0.508 ± 0.006), close to BHT (0.631 ± 0.008) and greater than quercetin (0.471 ± 0.008), indicating moderate antioxidant efficacy. At the longer duration of 120 min, EOLF reached 0.397 ± 0.001, confirming a significant protective effect on β-carotene and a plateau of activity close to BHT (0.496 ± 0.003) and greater than quercetin (0.278 ± 0.003). In terms of final inhibition, EOLF achieved a mean efficacy of 80% inhibition, which was like that of BHT (100%) and greater than quercetin (61%), while the untreated control showed only 22% inhibition. These results confirm that EOLF has significant fat-soluble antioxidant capacity, capable of effectively protecting β-carotene from oxidation. This activity is probably due to the synergistic action of the phenolic and terpinenes present in the essential oil, such as carvacrol and cis-sabinene.

### 2.3. Antimicrobial Activity

**Figure 6:**
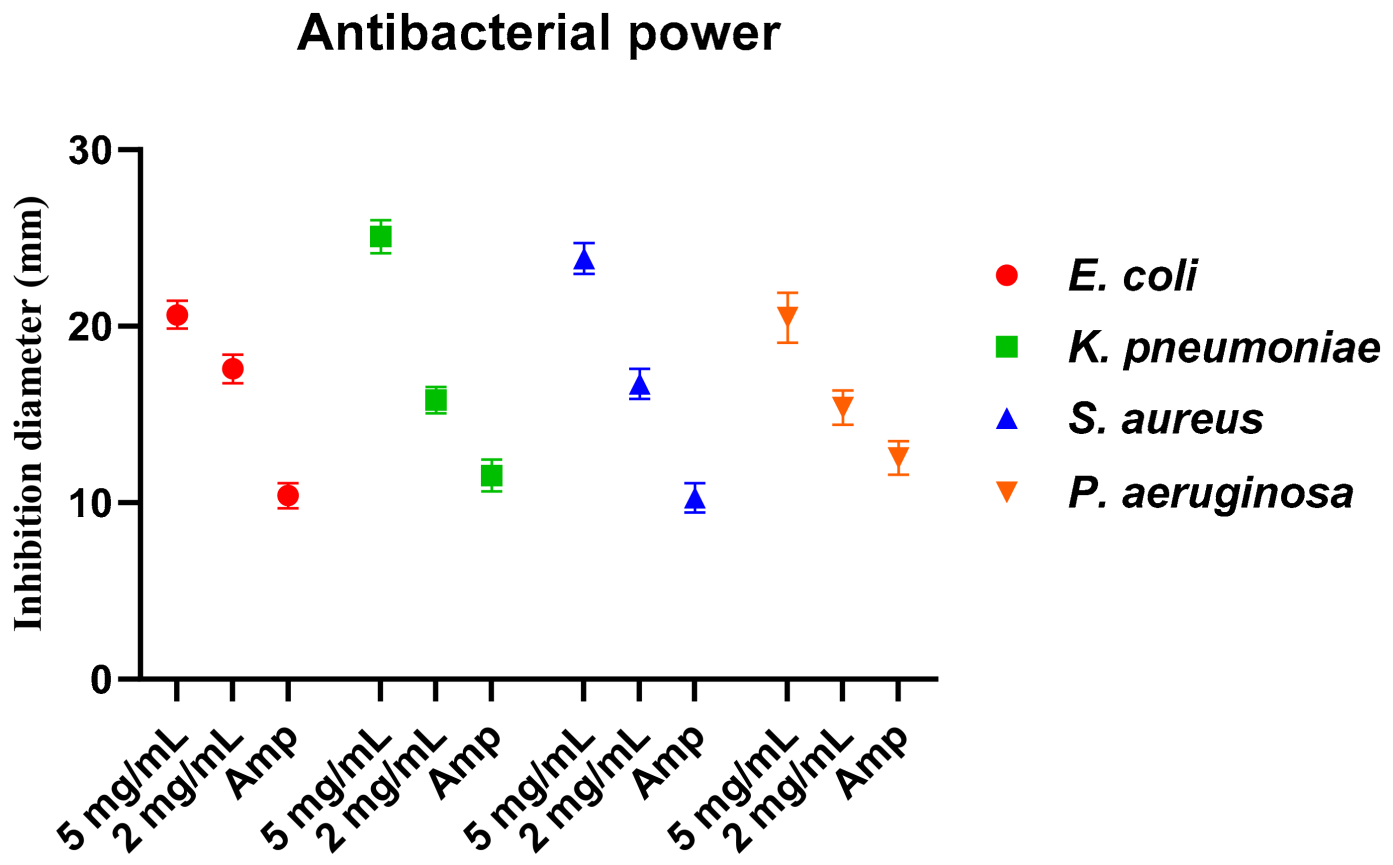
Antibacterial activity of *L. ferocissimum* essential oil (EOLF) evaluated on solid medium using the disc diffusion method.

The widths of the inhibition zones expanded with higher EOLF concentrations for all examined bacterial strains, indicating a concentration-dependent antibacterial action (Figure 6). At a concentration of 5 mg/mL, EOLF showed maximum inhibition zones ranging from 20.48 ± 1.41 mm to 25.08 ± 0.93 mm, depending on the bacterial strain. The most marked activity was observed against *K. pneumoniae* (25.08 ± 0.93 mm), followed by *S. aureus* (23.84 ± 0.88 mm), while *E. coli* and *P. aeruginosa* showed slightly lower zones (20.65 ± 0.78 mm and 20.48 ± 1.41 mm, respectively). At 2 mg/mL, antibacterial activity remains significant, albeit attenuated, with zones ranging from 15.39 ± 0.98 mm (*P. aeruginosa*) to 17.58 ± 0.82 mm (*E. coli*). These results indicate a dose-dependent relationship between essential oil concentration and inhibition of bacteria. In comparison, the standard antibiotic ampicillin (Amp) had smaller inhibition zones, ranging from 10.28 ± 0.84 mm (*S. aureus*) to 12.54 ± 0.94 mm (*P. aeruginosa*), which indicates weaker antibiotic activity than EOLF. This difference suggests that the essential oil is remarkably effective, particularly against Gram-negative strains including *E. coli*, *K. pneumoniae*, and *P. aeruginosa*, which are often resistant to conventional antibiotics.

The results reported in the literature indicate that extracts of *L. barbarum* and *L. chinense* exhibit moderate to greater antibacterial activity, varying according to the bacterial strains tested. The reported inhibition zones are generally between 12 and 24 mm, with more pronounced efficacy against Gram-positive bacteria such as *Staphylococcus aureus*, *Bacillus subtilis* and *Listeria monocytogenes* [14]. In particular, *L. chinense* was more sensitive to the antibacterial activity of EOLF than *L. barbarum*, with inhibition zones reaching 24.2 ± 0.6 mm against *Bacillus subtilis* and 21.6 ± 0.8 mm against *Listeria monocytogenes*. In contrast, Gram-negative bacteria, such as *E. coli* and *S. typhimurium*, were more moderately sensitive, with inhibition zones ranging from 12.3 ± 0.8 mm to 19.6 ± 0.3 mm, which is generally attributed to the more complex structure of their cell wall [14].

Overall, data from the literature indicate that the antibacterial activity of *Lycium* species is dependent on their phytochemical composition, particularly the presence of phenolic compounds and other bioactive metabolites capable of disrupting the integrity of bacterial membranes. In this context, these observations corroborate the strong broad-spectrum antibacterial potential of EOLF, which could be attributed to its richness in phenolic monoterpenes such as carvacrol, as well as oxygenated compounds, particularly borneol, cis-sabinene and caryophyllene oxide, known for their ability to alter membrane permeability and lyse bacterial cells.

When the antibacterial activity of EOLF in liquid medium was evaluated by determining the MIC (µg/mL), corresponding to the lowest concentration capable of completely blocking bacterial growth, EOLF effectively inhibited all strains tested, with MICs ranging from 11.03 ± 0.27 µg/mL for *S. aureus* to 16.29 ± 0.79 µg/mL for *P. aeruginosa* (Table 2). The Gram-positive bacterium *S. aureus* appears to be the most sensitive, suggesting that EOLF acts effectively on its cell wall and may disrupt its essential metabolic processes. Gram-negative bacteria (*E. coli*, *K. pneumoniae*, *P. aeruginosa*) require slightly greater concentrations for complete inhibition, which could be explained by the presence of their protective outer membrane, limiting the penetration of bioactive compounds. Compared to streptomycin (Stpt), EOLF shows similar efficacy depending on the strain, indicating its potential as a natural alternative to synthetic antibiotics. The negative control showed no inhibition (0.0 ± 0.0 µg/mL), confirming the specificity and reliability of the results.

The MIC values reported in the literature confirm moderate antibacterial activity of bioactive compounds extracted from plants of the *Lycium* genus, particularly *Lycium barbarum* and *Lycium chinense*, with MICs generally ranging from 50 to >100 µg/mL depending on the bacterial strain. In general, *L. chinense* has lower MIC values than *L. barbarum*, which is consistent with greater antibacterial efficacy. For *S. aureus* and *L. monocytogenes*, both extracts show limited efficacy, with MIC values above 100 µg/mL, suggesting relative resistance of these bacteria to the active compounds present in the extracts. In contrast, *Bacillus subtilis* and *E. coli* appear to be more sensitive, with MIC values of 100 µg/mL for *L. barbarum* and 75 µg/mL for *L. chinense*. The most sensitive strain remains *Salmonella typhimurium*, for which the lowest MICs were observed, reaching 75 µg/mL for *L. barbarum* and 50 µg/mL for *L. chinense*. This increased sensitivity could be related to better interaction of the bioactive metabolites with the bacterial cell membrane [14]. Overall, these results from the literature indicate that the phenolic compounds of *L. chinense* have greater antibacterial potential than those of *L. barbarum*. The variations observed between bacterial strains and between the two plant species highlight the importance of specific phytochemical composition, as well as bacterial cell wall structure, in determining antibacterial activity. In comparison, EOLFs exhibit significantly stronger antibacterial activity, resulting in lower minimum inhibitory concentrations than those reported for *L. barbarum* and *L. chinense*. This superiority could be explained by their content of bioactive terpenic compounds, which promote more effective diffusion through bacterial membranes and more intense inhibitory action. This antibacterial activity is mainly attributed to the presence of phenolic and terpenic compounds, such as carvacrol, γ-terpinene and cis-sabinen, known for their ability to destabilize the bacterial cell membrane, alter its permeability and induce leakage of intracellular content, thus leading to the death of bacterial cells.

**Figure 7:**
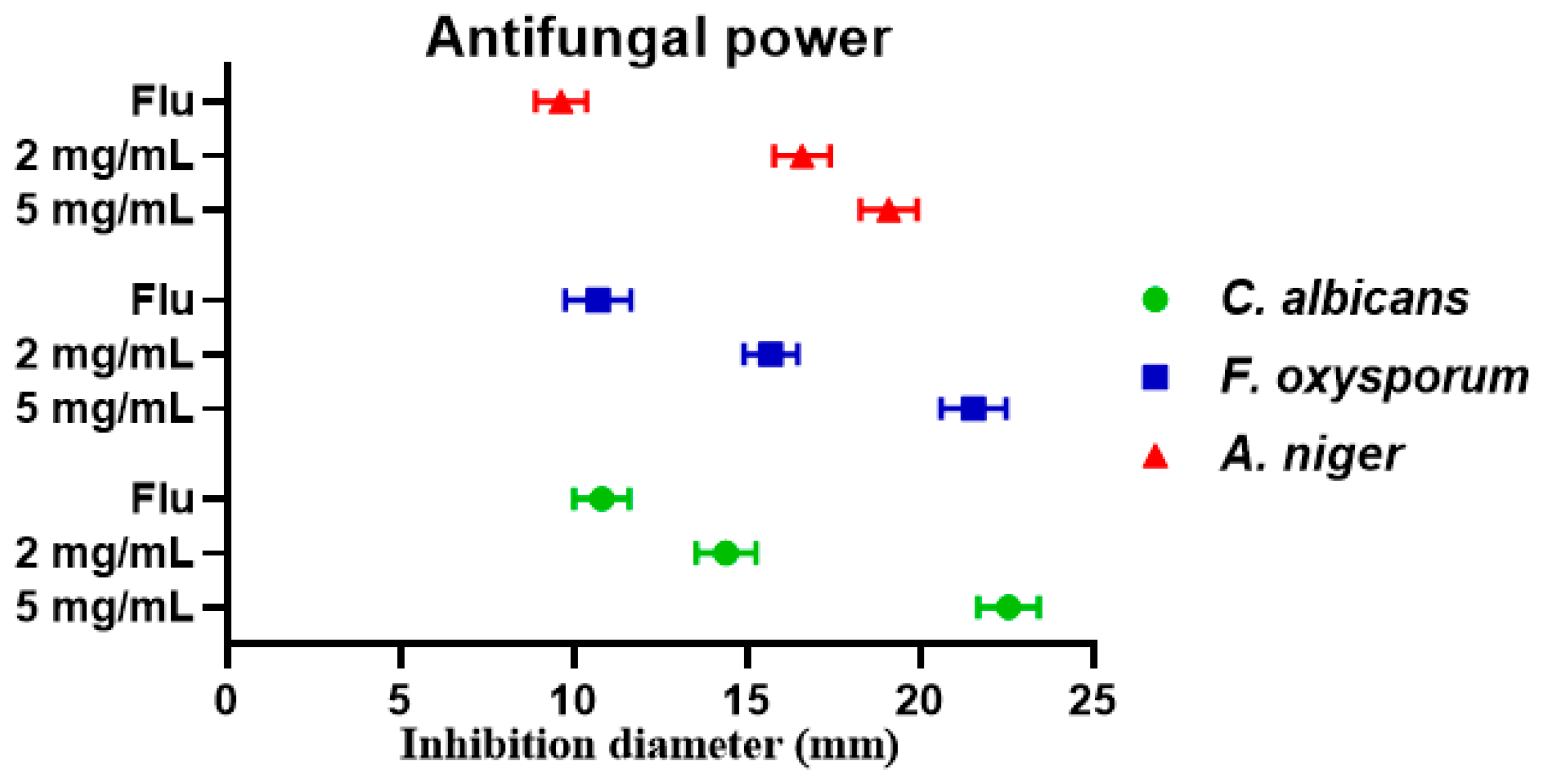
Antifungal activity of *L. ferocissimum* essential oil (EOLF) evaluated on solid medium using the disc diffusion method.

When the antifungal activity was evaluated on solid medium using the agar diffusion method, which measures the diameter of the inhibition zones for *C. albicans*, *F. oxysporum* and *A. niger*, EOLF exhibited strong antifungal activity, which was concentration-dependent (Figure 7). At 5 mg/mL, the inhibition zones recorded were 22.55 ± 0.87 mm for *C. albicans*, 21.54 ± 0.94 mm for *F. oxysporum*, and 19.09 ± 0.82 mm for *A. niger*. These values demonstrate significant antifungal efficacy, which was superior to that of fluconazole, used as a reference antifungal agent (10.81 ± 0.80 mm, 10.70 ± 0.95 mm and 9.63 ± 0.74 mm, respectively). At 2 mg EOLF/mL, although activity decreased slightly, inhibition was significant with zones ranging from 14.39 ± 0.86 mm to 16.59 ± 0.81 mm, confirming a dose-dependent relationship between concentration and antifungal potency. This activity on solid media can be attributed to the synergy between several major compounds in the essential oil, such as carvacrol, γ-terpinene, p-cymene and cis-sabinene, which are known for their antifungal properties. These molecules act mainly by altering the permeability of the cell membrane, causing the leakage of intracellular constituents and interfering with the mitochondrial respiration of fungi. Thus, EOLF has promising antifungal potential, capable of effectively inhibiting various pathogenic fungal genera on solid media. These results suggest that this essential oil could be a natural alternative or a potential adjuvant to synthetic antifungals in the fight against resistant fungal infections.

EOLF oil has also demonstrated strong antifungal potential in liquid media, as assessed by determining MICs. The MICs obtained ranged from 10.51 ± 0.47 µg/mL for *C. albicans* to 12.88 ± 0.93 µg/mL for *A. niger*, indicating significant and effective activity (Table 2). Among the strains tested, *C. albicans* was the most sensitive, followed by *F. oxysporum* and *A. niger*, which suggested that EOLF has a particular effect against pathogenic yeasts and moulds. Comparison with fluconazole (positive control) shows that EOLF has an efficacy close to that of this antifungal standard, which is remarkable for a natural product. This antifungal activity is probably linked to the lipophilicity and reactivity of the phenolic and terpenic compounds present in the essential oil, particularly carvacrol, p-cymene and cis-sabinene, which interfere with fungal cell membranes and disrupt essential enzymatic functions, leading to growth inhibition and cell death. Thus, the results obtained in liquid medium confirm that EOLF has a broad spectrum of antimicrobial activity, effective even at low concentrations, and is a promising natural source for the development of antibacterial and antifungal products.

The presence of carvacrol, identified as one of the main constituents of EOLF, could contribute significantly to its antimicrobial activity. Indeed, Mączka et al. (2023) reported notable antimicrobial activity of carvacrol against various bacterial and fungal species, notably *Candida albicans* [15]. This activity is notably attributed to its ability to disrupt the integrity and permeability of cell membranes, thereby leading to impaired cellular functions. The high carvacrol content in EOLF could therefore contribute, at least in part, to the antibacterial and antifungal activities observed in the present study. More broadly, plant-derived secondary metabolites may act via several complementary mechanisms, notably by disrupting cell membranes, inhibiting efflux pumps and limiting biofilm formation, which could explain their potential efficacy against multidrug-resistant microorganisms [16]. In the same context, Rafiq et al. identified several bioactive constituents in an extract of Carthamus oxycantha using GC-MS, some of which exhibited antibacterial and antifungal properties [17]. These observations highlight the potential of plant-derived compounds as sources of natural antimicrobial agents and reinforce the significance of the constituents identified in the EOLF in explaining, at least in part, the observed antimicrobial activity [17].

### 2.4. In-Silico Studies

#### 2.4.1. In-Silico Drug-Likeness and Pharmacokinetic ADMET Prediction

In the context of drug development, it is crucial to anticipate the effects of a compound on the body, such as the amount absorbed through the digestive system when administered orally and its final destination within the organism. Inadequate absorption may affect the distribution and metabolism of a drug, thereby increasing the risk of toxicity. To assess these initial bioavailability and safety criteria, the well-known Lipinski’s Rule of Five, which predicts the drug-like properties of candidate molecules, establishes the following standards: the number of hydrogen bond donors should not exceed 5, the number of hydrogen bond acceptors should not exceed 10, the molecular weight should not exceed 500 Da, and the octanol/water partition coefficient (log P) should not be greater than 5. As mentioned, if a compound violates two or more of Lipinski’s rules, it is generally not considered orally active. None of the proposed compounds violated Lipinski’s Rule of Five [18]. Regarding the parameters, ADMET intestinal absorption determines the percentage of a drug absorbed through the digestive system, which is often the predominant route of drug absorption, and an absorption rate below 30% is considered low. However, the results revealed that all compounds exhibited good absorption in the human intestine (Table S1). However, these in silico predictions require experimental validation to confirm the predicted intestinal absorption profile.

Responses of the human intestinal mucosal cell line are considered in this parameter. Caco-2 cells are commonly used as an in vitro model to predict the absorption of orally administered drugs. If the pkCSM model predicts a compound with a logPapp value (10^−6^ cm/s) greater than 0.90, it is classified as having great Caco-2 cell permeability. All constituents’ compounds met the Caco-2 permeability criterion. The volume of distribution (Vd) is a pharmacokinetic parameter that illustrates the tendency of a compound to remain in plasma or distribute into other tissues. A VDss is considered *di minimis* when it is less than 0.71 L/kg (log VDss < −0.15) and great when it exceeds 2.81 L/kg (log VDss > 0.45) [19]. According to the analyzed data, a significant difference in Vdss values was observed among the compounds: some exhibited intermediate distribution (between −0.15 and 0.45), whereas several showed extensive distribution (log Vdss > 0.45).

The blood–brain barrier (BBB) is a selective barrier that restricts the access of many drugs to the central nervous system (CNS). To potentially avoid adverse effects, drugs designed to treat other parts of the body should not cross the BBB. However, all evaluated compounds showed BBB values ranging from 0.381 to 0.837, suggesting CNS permeability. Metabolism was estimated based on CYP models regarding their inhibitory role toward CYP2D6 and CYP3A4. Cytochrome P450 (CYP450) isoenzymes are oxidases that modify drugs to reduce their plasma concentration and limit toxicity risks through metabolic activation, while increasing their water solubility to facilitate elimination. Therefore, a drug candidate should not inhibit CYP450 isoenzymes, since such inhibition may lead to an increase in plasma concentration. Table 1 indicates that the compounds are not inhibitors of CYP450 2D6 and 3A4 isoenzymes, suggesting the absence of a significant risk of drug interactions mediated by these CYPs. Total clearance values indicate poor to low elimination for all compounds, suggesting that these molecules may remain in the body for an extended period. Hepatotoxicity refers to the impairment of normal liver function, and the predicted values indicated that none of the compounds exhibited potential liver toxicity [20]. Ames test predictions showed that the compounds are non-mutagenic. Overall, these results confirm the reliability of the docking protocol used in this research. In summary, the LD50 estimates ranged from 1.528 to 2.016 mol/kg. Cis-sabinene and Caryophyllene oxide had the lowest predicted LD50 values, at 1.528 and 1.534 mol/kg, respectively, suggesting a slightly higher presumed acute toxicity than the other compounds examined. Conversely, Isoborneol demonstrated the highest estimated LD50 value (2.016 mol/kg), suggesting the lowest presumed acute toxicity potential among the compounds studied. Typically, relatively high LD50 estimates suggest a presumed beneficial acute toxicity. This demonstrated that the compounds would only be lethal at very great concentrations, indicating low acute toxicity, and would not be expected to elicit toxic effects at therapeutic doses.

#### 2.4.2. Molecular Docking of Proposed Compounds

The molecular docking method was used to determine the binding geometries as well as the interactions between the proposed compounds and the active site of a protein [21]. To identify their binding modes and ligand–protein interactions, molecular docking studies of these compounds were conducted to predict interactions with key bacterial and fungal proteins (Table 3). The docking protocol was validated by a redocking procedure, and the RMSD values between the experimental and redocked ligand poses were determined. The obtained RMSD values ranged from 0.398 to 2.058 Å, with values below the commonly accepted threshold of 2.0 Å for six of the seven investigated proteins, including 0.920 Å for 3G7B, 0.793 Å for 1EAG, 0.873 Å for 6Z5K, 0.663 Å for 8EBB, 0.398 Å for 7BLY, and 1.055 Å for 8JGW. For 1KZN, an RMSD value of 2.058 Å was obtained, which is very close to the accepted threshold. The grid center coordinates (x, y, z) were set to (50.354241, −2.963621, 19.128759) for 3G7B, (41.694642, 24.906453, 13.626925) for 1EAG, (94.325244, 46.196415, 9.072659) for 6Z5K, (19.150163, 30.392531, 34.744776) for 1KZN, (20.239700, 9.382200, −34.827400) for 8EBB, (23.407520, 53.867449, −20.760931) for 7BLY, and (−20.278500, −6.800167, 6.656500) for 8JGW. Overall, these results attest to the reliability of the docking protocol used in this research.

**Figure 8:**
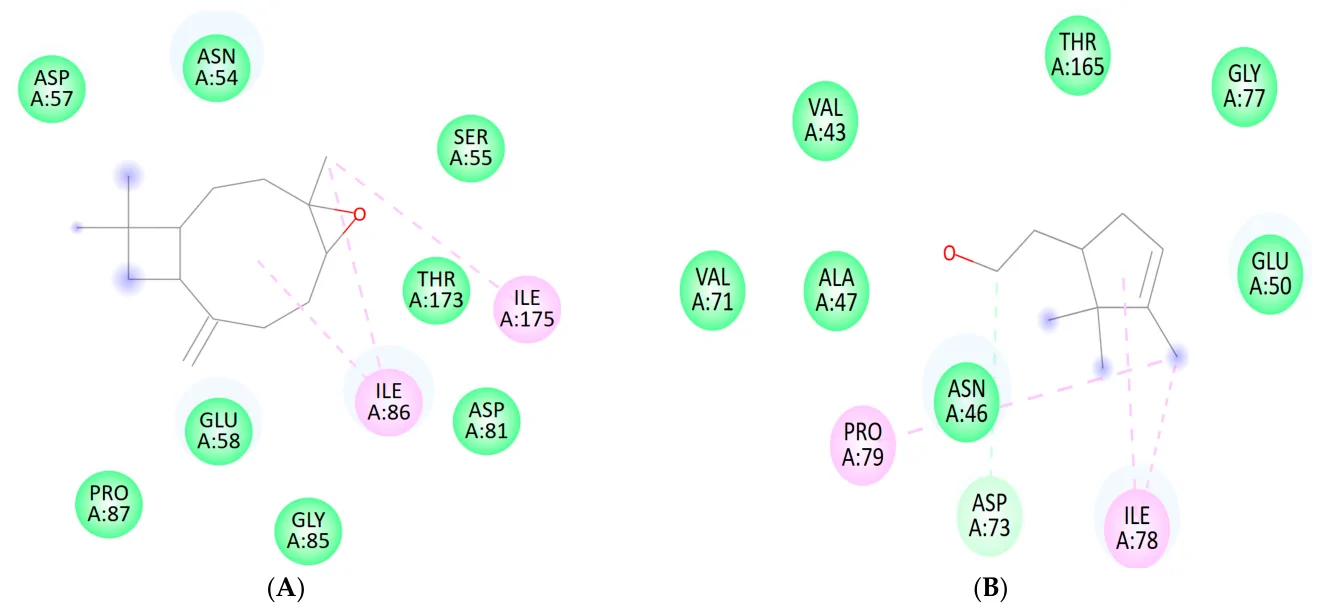

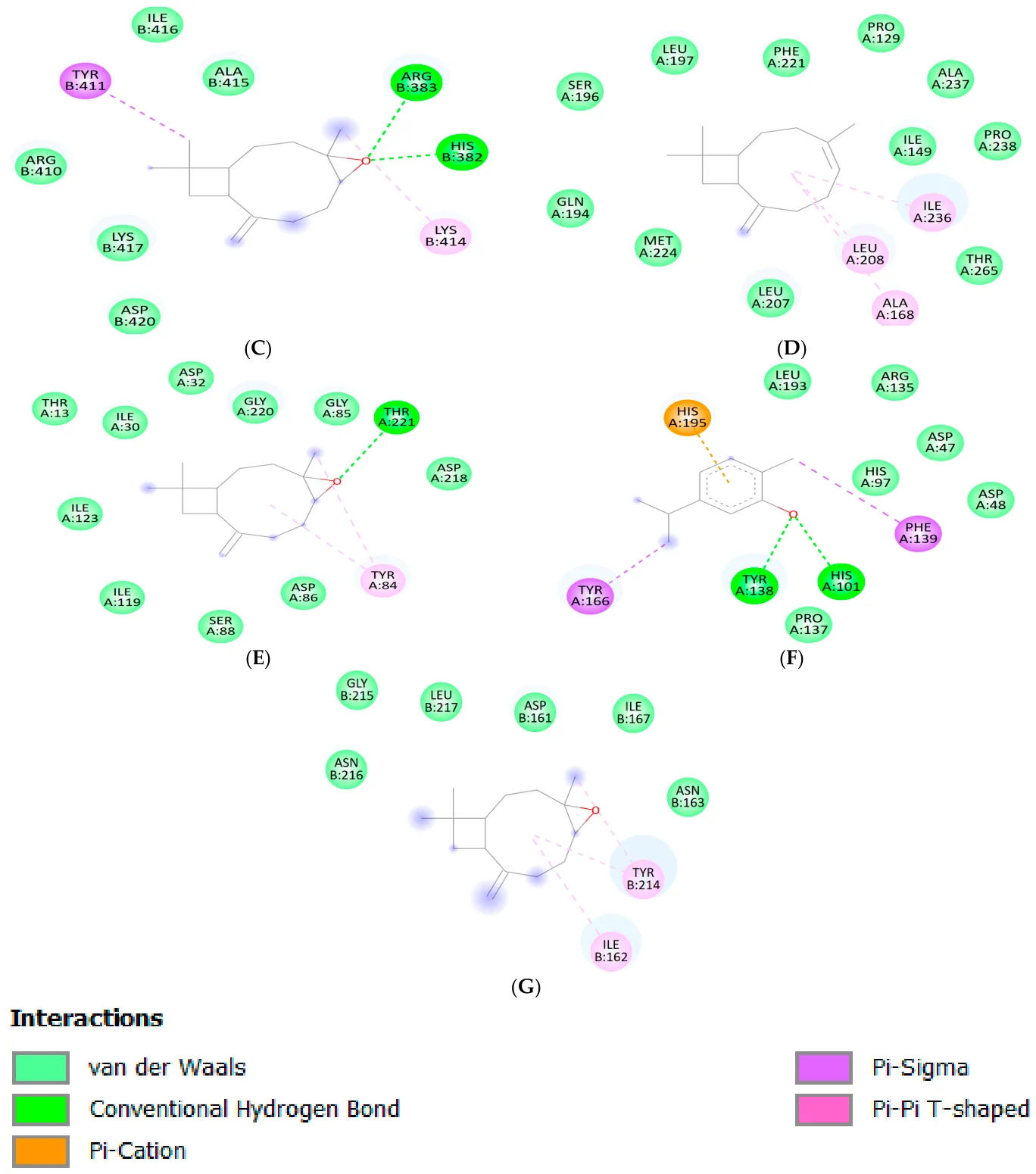
Protein–compound interaction complexes (EOLF): (**A**) *3G7B*–caryophyllene oxide, (**B**) *1KZN*–α-campholenol, (**C**) *8JGW*–caryophyllene oxide, (**D**) *6Z5K*–caryophyllene, (**E**) *1EAG*–caryophyllene oxide, (**F**) *7BLY*–carvacrol, (**G**) *8EBB*–caryophyllene oxide.

The molecular docking results revealed that hydrophobic interactions, as well as Alkyl and van der Waals forces, played a predominant role in interactions between constituent chemicals of MOLF and most target proteins. Hydrogen bonds were also identified as important contributors to the interactions within some complexes. Interactions of the compound exhibit a strong affinity toward the *3G7B* target (Figure 8A). Caryophyllene oxide demonstrated a binding capacity of −6.7 kcal/mol, surpassing that of the reference drug, with hydrophobic interactions involving the residues ILE86 and ILE175. Regarding the *1KZN* target protein, the compound α-campholenol exhibited hydrophobic interactions with PRO79 and ILE78, as well as a hydrogen bond with the amino acid ASP73, showing a binding affinity of −5.2 kcal/mol comparable to that of streptomycin (Figure 8B). The residues of the *8JGW* protein interacting with Caryophyllene oxide were TYR411 and LYS414, and two hydrogen bonds with HIS382 and ARG383 were also observed, presenting a binding affinity of −6.4 kcal/mol (Figure 8C). The compound Caryophyllene interacted with ILE236, LEU208, and ALA168, displaying a binding affinity of −8.0 kcal/mol, which was greater than that of streptomycin toward the *6Z5K* target protein (Figure 8D). Caryophyllene oxide exhibited a remarkable binding affinity of −7.1 kcal/mol, surpassing the binding affinity of fluconazole, which was lower in comparison with Caryophyllene oxide against the *1EAG* protein (Figure 8E). Its compatibility was demonstrated by the formation of two hydrophobic interactions with residue TYR84 and one hydrogen bond with residue THR221. The great docking score of −6.3 kcal/mol for the Carvacrol–*7BLY* complex was attributed to the formation of two hydrogen bonds with residues HIS101 and TYR138, as well as C–H interactions with residues PHE139 and TYR166. Another π–cation interaction was also involved with residue HIS195 (Figure 8F). The interaction of Caryophyllene oxide with *8EBB* was stabilized by strong hydrophobic interactions with residues TYR214 and ILE162, characterized by a binding affinity of −5.5 kcal/mol (Figure 8G).

#### 2.4.3. Molecular Dynamics Results Analysis

To assess their stability, the compounds that exhibited the best scores with their target proteins were selected for molecular dynamics studied. Descriptors such as RMSD and RMSF curves, as well as interaction diagrams, were used. Regarding the *S. aureus*–caryophyllene oxide complex, the protein 3G7B shows overall stability with an RMSD around 1.2 Å, while the ligand maintains a stability of approximately 1.5 Å throughout the simulation. A slight variation of up to 2 Å is observed at the end of the trajectory; however, this variation remains minimal and does not affect the overall stability of the complex. This behavior indicates a balanced and stable system (Figure S1A) [22]. The *1KZN*–α-campholenol complex exhibits protein *E. coli* stability, showing an approximate RMSD of 2.1 Å throughout the simulation. However, the ligand displays a greater RMSD, of approximately 5.6 Å, which indicated a significant conformational rearrangement of the ligand within the binding site, while remaining generally bound to the system (Figure S1B). For the *8JGW*–caryophyllene oxide interaction, a notable increase in protein *K. pneumoniae* RMSD was observed, rising from approximately 2.4 Å to 4.8–5.6 Å after 20 ns, reflecting a gradual decrease in structural stability. As for the ligand, a drift of about 17.8 Å is observed, suggesting a probable detachment from the active site and an overall instability of the complex (Figure S1C). The *6Z5K*–caryophyllene complex exhibited moderate stability, with variations ranging from 3 Å to 3.5 Å. The ligand exhibits more pronounced fluctuations between 4 Å and 5.6 Å, suggesting some flexibility but without complete dissociation from the protein (Figure S1D). Overall, the *1EAG*–caryophyllene oxide complex appears to maintain consistent stability. The protein *C. albicans* shows moderate variations ranging from 2.5 Å to 3.5 Å, while the ligand maintains relatively stable behavior around 5 Å, indicating the dynamic coherence of the system (Figure S1E). In contrast, the *7BLY*–carvacrol complex demonstrates unstable behavior. Although the protein *A. niger* shows relatively low RMSD values, it undergoes successive conformational changes, while the ligand exhibits significant instability with large displacements reaching as much as 60 Å, suggesting a complete detachment from the active site (Figure S1F) [23]. Finally, the *8EBB*–caryophyllene oxide complex presents a stable protein with an approximate RMSD of 1.4 Å, indicating a well-preserved and robust structure. However, the ligand exhibits multiple conformational states, fluctuating around 1.2 Å, 2.4 Å, and 4.8 Å, indicating a dynamic adjustment of the ligand within the binding cavity without complete loss of interaction (Figure S1G).

The evaluation of the local flexibility of amino acids in the protein structure during the simulation is made possible through the use of the root mean square fluctuation of residues (RMSF) [24]. Great values reflect highly flexible regions, whereas low values are associated with structured and stable zones. As shown in Figure S2, all examined complexes, even those exhibiting large variations in terms of RMSD, reveal generally low RMSF values for most residues. These values remain below ~3 Å, suggesting that the protein structure maintains fairly uniform local stability throughout the simulation. However, this local residue stability is not necessarily representative of the overall stability of the protein–ligand complex [25]. Indeed, some complexes with significant RMSD variations still show RMSF profiles comparable to those of stable complexes, which suggests that the detected fluctuations mainly affect the global position of the ligand or the complex rather than the flexibility of protein residues. Thus, the analysis of system stability cannot be based exclusively on RMSF. Additional analyses of protein–ligand interactions (hydrogen bonds, hydrophobic interactions, ionic interactions, and hydrophobic contacts) are necessary in order to assess the persistence of the binding mode and to validate the actual stability of the complexes during the simulation.

The study of protein–ligand interactions performed during molecular dynamics reveals variations among the seven analyzed complexes, particularly regarding the stability of hydrogen bonds and hydrophobic interactions. For the *3G7B*–caryophyllene oxide complex, a predominant hydrogen bond interaction is observed with residue ASN54, showing a large rate of occupancy of approximately 83%, complemented by hydrophobic interactions involving, among others, ILE86 and ILE108 at around 25%. This combination of stable interactions indicates a robust binding of the ligand to the active site, contributing to the overall stability of the complex (Figure S3A). Similarly, the *1KZN*–α-campholenol complex shows a strongly preserved hydrogen bond interaction with residue ASP73, with an occupancy of approximately 88%. This persistent bond confirms a crucial contact that maintains the ligand within the binding cavity, also indicating a highly stable profile (Figure S3B) [26]. In contrast, the *8JGW*–caryophyllene oxide complex does not exhibit significant interaction, whether hydrogen or hydrophobic, with all interactions less than 0.06%. This lack of stable interactions indicates insufficient retention of the ligand in the active site, consistent with the unstable behavior observed during the simulation (Figure S3C). The *6Z5K*–caryophyllene complex is mainly maintained by hydrophobic interactions involving various residues, including ALA102, PRO129, ILE149, LEU197, LEU208, ILE236, and PRO238, with contributions ranging from 0.11% to 0.17%. Although these individual values are relatively low, the large number of hydrophobic contacts indicates an extended stabilization of the ligand within the binding site (Figure S3D). The *1EAG*–caryophyllene oxide complex exhibits a dominant hydrophobic interaction with residue TYR84 exceeding 0.20%, while other residues, such as VAL12, ILE30, ILE119, and ILE123, show lower contributions. This distribution reflects a moderately hydrophobic network, with TYR84 playing a central role in ligand binding (Figure S3E). In contrast, the *7BLY*–carvacrol complex shows very minimal interactions, all less than 0.05%, with no major interaction identified. This absence of sustained contacts indicates reduced ligand stability in the binding site and an unfavorable interaction with the protein (Figure S3F). Finally, the *8EBB*–caryophyllene oxide complex was characterized by more diverse and relatively stable interactions, including a water-mediated hydrogen bond with GLY85 (0.45%), a hydrophobic contact with ILE86 (0.38%), and a significant interaction with ASP81 (0.9%). The combination of these interactions, including the presence of a stabilizing water bridge, suggests a more organized and potentially more durable binding mode compared to complexes 3 and 6 (Figure S3G).

## 3. Materials and Methods

### 3.1. Plant Identification

**Figure 9:**
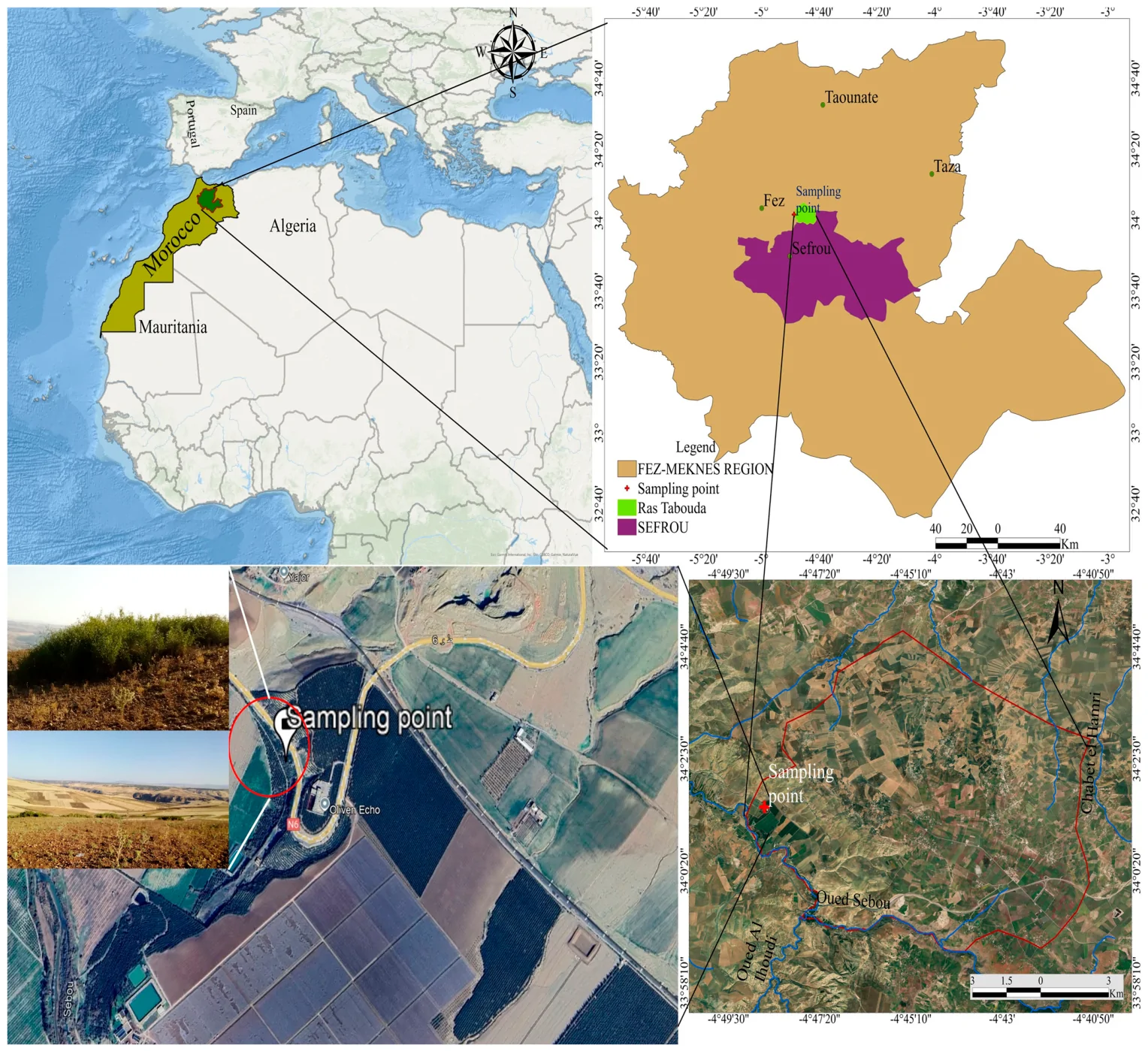
Location from which *L. ferocissimum* was harvested.

Leaves of *L. ferocissimum* were harvested in April 2025 from an arid and semi-arid climate region (Figure 9), where the plant is naturally adapted to desert conditions and tolerates environmental stress. Plants were collected from a rural agricultural area (34.025643; −4.810036), where the species is often associated with other thorny plants such as jujube, in disturbed habitats. Specimens were identified by Professor El Moussaoui Abdelfattah, and a type specimen was deposited in the biology department’s herbarium under voucher number AEM-FS/T/LF01/2025.

### 3.2. Preparation of Plant Material

Fresh leaves of *L. ferocissimum* were collected, cleaned to eliminate any impurities, and then dried in the dark in an oven at 40 °C. Once dried, they were ground into a fine powder using an electric blender. The resulting powder was stored in tinted glass vials (15 °C) for the subsequent extraction of bioactive compounds.

### 3.3. Essential Oil Extraction

EOLF was extracted from leaves by hydrodistillation using a Clevenger-type apparatus connected to a 1000 mL flask. Specifically, 200 g of dried *L. ferocissimum* leaf powder was added to 750 mL of distilled water, and the mixture was boiled for 180 min. At the end of the extraction, the essential oil obtained was collected and carefully stored at 4 °C until it was used for further analysis [27].

### 3.4. GC-MS Identification and Quantification

ELOF was analyzed using GC-MS on a TQ8040 NX chromatograph (Shimadzu, Tokyo, Japan) coupled with a triple quadrupole tandem mass spectrometer. The compounds were separated on an RTxi-5 Sil MS non-polar column (30 m × 0.25 mm ID × 0.25 µm; Shimadzu, Tokyo, Japan), using high-purity helium as the carrier gas and an injection volume of 1 µL. The spectrometer source temperature was maintained at 200 °C. Injection was performed without initial splitting, which only occurred after 4 min. The injector temperature was set at 250 °C with a constant pressure of 37.1 kPa. The heating program started at 50 °C for 2 min, and then increased at a rate of 5 °C/min to 160 °C, which was maintained for 2 min, before continuing at 5 °C/min to 280 °C for 2 min. The compounds were identified by comparing their mass spectra with those available in the NIST database and in Adams (2007), as well as by comparing their calculated retention indices (RIs) [28,29].

### 3.5. Antiradical Activity

Antioxidant potency was assessed in vitro by using four methods, including DPPH, FRAP, TAC and the *β*-carotene/linoleic acid test. These four approaches measure the ability of samples to trap free radicals or reduce oxidized metal ions. They were applied to both the essential oils studied and the reference antioxidants, BHT (butylhydroxytoluene) and quercetin, applied as positive controls.

#### 3.5.1. DPPH Assay

ELOF was diluted in methanol at a ratio of 1:20 (*v*:*v*), and then a series of successive dilutions was prepared in the same solvent [30]. At the same time, a DPPH solution was prepared in methanol at a concentration of 4%. The antioxidant capacity was assessed by mixing 100 µL of each essential oil dilution with 750 µL of the DPPH solution. The mix was incubated for 30 min at room temperature, and then the optical density (OD) was measured using a UV spectrophotometer at a wavelength of 512 nm. The antioxidant capacity of the samples was determined by comparing the concentrations inhibiting 50% of free radicals (IC_50_). The percentage inhibition was calculated (Equation (1)) [31].

Inh (%) = [1 − Ae/At] × 100,(1)

where **Inh (%)**: percentage inhibition; **Ae**: optical density of the sample studied (EO, BHT and Quercetin); **At**: optical density of negative control.

#### 3.5.2. FRAP Assay

For the FRAP test, 500 µL of phosphate buffer (0.2 M, pH 6.6), 500 µL of potassium ferricyanide (1%) and 100 µL of different concentrations of samples dissolved in methanol were mixed. The mixture was then incubated at 50 °C for 20 min to allow the reduction of ferricyanide (Fe^3+^) to ferrocyanide (Fe^2+^) [32]. After this step, 500 µL of an aqueous solution of TCA (10%) was added to stop the reaction and precipitate the proteins. Next, 500 µL of distilled water and 100 µL of FeCl_3_ solution (0.1%) were added to promote the formation of the Prussian blue complex (Fe^4+^[Fe^3+^(CN)_6_]_3_), the intensity of which is proportional to the reducing capacity of the sample. The absorbance of the reaction mix was determined at 700 nm using a UV–visible spectrophotometer. An increase in absorbance indicates a greater iron reduction capacity and, consequently, a greater antioxidant capacity. The results are expressed as the 50% effective concentration (EC_50_), defined as the concentration of EOLF corresponding to an optical density (OD) of 0.5, as determined from the concentration–OD response curve. The lower the EC_50_ value, the greater the antioxidant power of the sample.

#### 3.5.3. TAC Assay

For the TAC test, 25 µL of each sample studied was added to a reagent solution composed of 0.6 M sulphuric acid (H_2_SO_4_), 28 mM sodium phosphate (Na_2_HPO_4_) and 4 mM ammonium molybdate ((NH_4_)_2_MoO_4_) [33]. The resulting reaction mixture was incubated in a water bath at 95 °C for 90 min, which promotes the reduction of molybdate (VI) to molybdate (V) under the action of the antioxidant compounds present in the sample. This reduction leads to the formation of a green phosphomolybdenum complex, the intensity of which is proportional to the reducing capacity of the sample. After cooling to room temperature, the absorbance of the mixture was measured at 695 nm using a UV–visible spectrophotometer. The total antioxidant capacity was determined by comparing the absorbance values obtained with a calibration curve established using ascorbic acid as a standard. The results are expressed in micrograms of ascorbic acid equivalents per milligram of sample (µg EAA/mg). This method allows the overall antioxidant power of essential oils to be evaluated based on their ability to reduce metal ions, in a manner comparable to that of ascorbic acid under similar experimental conditions.

#### 3.5.4. β-Carotene Assay

This method is based on the preparation of a *β*-carotene/linoleic acid emulsion, which allows the antioxidant capacity of samples to inhibit *β*-carotene oxidation to be evaluated [34]. To do this, 0.1 mg of *β*-carotene was dissolved in 10 mL of chloroform. Subsequently, 1 mL of this solution was added to a mixture containing 100 mL of Tween 80 and 10 mL of pure linoleic acid. The chloroform was then completely evaporated using a rotary evaporator, and the dry residue obtained was dissolved in 25 mL of 30% hydrogen peroxide, thus forming the stock emulsion. From this emulsion, 2.5 mL was distributed into test tubes, to which either 100 µL of the sample under study or a standard solution of reference antioxidants (BHT and quercetin) was added. The initial absorbance was measured at 470 nm immediately after vigorous stirring of the mixture, comparing it to that of a blank without β-carotene. The tubes were then incubated in a water bath at 50 °C for three hours, and absorbance measurements were taken every 30 min to monitor the kinetics of *β*-carotene discoloration, which is indicative of antioxidant activity. A negative control was performed by replacing the test sample with 100 µL of methanol mixed with 2.5 mL of the β-carotene emulsion. The antiradical capacity was measured using the following mathematical formula [35]:

PA (%) = [As/Ac] × 100,(2)

**PA**: antiradicalic power; **As**: sample absorbance; **Ac**: positive control absorbance.

### 3.6. Antimicrobial Activity

#### 3.6.1. Antimicrobial Activity on Solid Medium

The antimicrobial action of EOLF was explored in vitro by applying the agar disc diffusion method with four bacterial strains, *Staphylococcus aureus* (LBMSA23), *Escherichia coli* (LBMEC22), *Klebsiella pneumoniae* (LBMKP23) and *Pseudomonas aeruginosa* (LBMPA20). Antifungal potency was assessed with three fungal strains, *Candida albicans* (LBMCA23), *Aspergillus niger* (LBMAA20) and *Fusarium oxysporum* (LBMFO22). The bacteria were grown on Mueller–Hinton (MH) medium, while the fungi were grown on Sabouraud Dextrose Agar (SDA) medium. The inocula were prepared from fresh cultures (18–24 h) and adjusted to a turbidity equivalent to the McFarland 0.5 standard (10^6^–10^8^ CFU/mL) in sterile saline solution (0.9% NaCl). For each test, 100 µL of the microbial suspension was incorporated into 5 mL of soft agar (0.5% agar-agar) maintained at 40–42 °C, and then poured onto plates containing the appropriate solid medium. After solidification, sterile filter paper discs (6 mm) were impregnated with 10 µL of essential oil and placed on the surface of the seeded medium. Positive controls were performed with streptomycin (30 µg/disc) for bacteria and fluconazole (15 mg/mL) for fungi, while a negative control was prepared with 5% DMSO solvent. The plates were incubated at 37 °C for 24 h for bacteria and at 30 °C for 48 to 72 h for fungi. After incubation, the diameters of the inhibition zones were measured in millimeters, and each test was performed in triplicate [36].

#### 3.6.2. Minimum Inhibitory Concentration (MIC)

The antimicrobial potency of EOLF was assessed employing the microdilution method to determine the MIC capable of blocking the growth of microorganisms in a liquid medium. For this test, 96-well microplates were used. In the first column, 100 µL of each sample diluted in 5% DMSO was deposited, while 50 µL of the appropriate liquid medium (Mueller–Hinton for bacteria and malt extract for fungi) was added to all wells. Next, 30 µL of the microbial suspension prepared from fresh cultures was added to each well. The microplates were incubated at 37 °C for 24 h for bacteria and at 30 °C for 48 to 72 h for fungi. The MIC was determined by adding 20 µL of 0.2% 2,3,5-triphenyltetrazolium chloride (TTC), which allows microbial growth to be visualized by a color change, with wells that remained colorless indicating complete growth inhibition [37].

### 3.7. In-Silico Study of Major Bioactive Compounds in EOLF

#### 3.7.1. Profiling ADMET

To predict the physicochemical and pharmacokinetic properties of the absorption, distribution, metabolism, excretion and toxicity (ADMET) of the major compounds detected in EOLF, the online servers SwissADME and pkCSM were consulted [38,39]. The SwissADME server was employed to assess bioavailability and to accurately evaluate the physicochemical properties, which should comply with Lipinski’s Rule of Five for safe and effective pharmaceuticals. In addition, the pkCSM web platform was primarily used to investigate the ADMET parameters of the targeted compounds.

#### 3.7.2. Docking, Analysis and Visualization

For this study, the 3D crystal structure of all the proteins involved was extracted in PDB format from the online Protein Data Bank (PDB) database. The targets chosen included proteins from *Staphylococcus aureus* (PDB ID: 3G7B; 2.30 Å), *Escherichia coli* (PDB ID: 1KZN; 2.30 Å), Klebsiella pneumoniae (PDB ID: 8JGW; 1.80 Å), *Pseudomonas aeruginosa* (PDB ID: 6Z5K; 3.20 Å), *Candida albicans* (PDB ID: 1EAG; 2.10 Å), *Aspergillus niger* (PDB ID: 7BLY; 1.81 Å) and *Fusarium oxysporum* (PDB ID: 8EBB; 1.88 Å). These proteins were selected to provide targets with specified structures that are relevant to the bacterial and fungal pathogens analyzed. The availability of experimentally determined structures facilitated the development of a more robust structural basis for molecular binding. Each structure was prepared by removing water molecules, heteroatoms, and the inactive chain, and by adding hydrogen atoms using AutoDock software version 1.5.7. Molecular docking was then performed, which is an essential tool in computational drug design, allowing the prediction of the main interaction mode of a ligand with a target protein. The compounds identified in the EOLF were optimized and then subjected to molecular docking analysis with the target proteins, with each protein treated as a macromolecule and the drug as a ligand. In this context, the reference drug was treated as the control, and the binding affinities of the compounds identified in the EOLF were compared with those of the reference drug. AutoDock Vina software version 1.1.2 was used to perform the flexible docking [40]. Finally, Accelrys Discovery Studio Visualizer version 21.1.0.20298 software was employed to examine and visualize the docking results [41].

#### 3.7.3. Molecular Dynamics Simulation

Compounds that achieved satisfactory in silico scores were subjected to molecular dynamics (MD) analyses using the Desmond tool of Maestro software version 2021-3. This enabled the examination of the dynamic behavior of protein–ligand complexes over time, based on a physical model that monitors interatomic interactions. The topology of the investigated ligand was generated using Discovery Studio software. Once the complex was imported, the OPLS3e force field [42] was employed for its minimization. The MD simulation model was built using the System Builder wizard, which incorporates the SPC water model. The system was enclosed in a periodic cubic box with a dimension of 10 Å. A neutral pH environment was achieved by adding counterions (Na^+^ and Cl^−^). MD simulations were conducted for a duration of 100 nanoseconds (ns), at a temperature of 300 Kelvin and a pressure of 1 bar [43].

### 3.8. Statistical Analysis

The findings from three separate experiments (n = 3) are shown as the mean ± standard deviation (SD). Prior to performing parametric statistical analysis, Levene’s test was used to evaluate the homogeneity of variances, and the Shapiro–Wilk test was used to verify the data’s normality. A one-way analysis of variance (ANOVA) was used to analyze group differences since the data satisfied the requirements for parametric study. Tukey’s multiple comparison test was then used. GraphPad Prism software (version 8) was used for statistical analyses; differences were deemed statistically significant at *p* < 0.05.

## 4. Conclusions

*L. ferocissimum* essential oil (EOLF) has significant antioxidant and antimicrobial potential, confirmed by results of various in vitro tests, including DPPH, FRAP, TAC, β-carotene and evaluation on solid and liquid media. EOLF exhibits dose-dependent activity, with relatively small IC_50_ and MIC values, similar to those of synthetic and other natural antioxidants, while EOLF had significant antioxidant effects at relatively small concentrations. Phytochemical analyses reveal the presence of major compounds such as carvacrol, cis-sabinene and γ-terpinene, which are responsible for the observed effects. The experimental outcomes were supported by the in-silico studies. The ADEM-Tox properties demonstrated that the compounds would only be lethal at very great concentrations, indicating low acute toxicity and would not be expected to elicit toxic effects at therapeutic doses. In addition, molecular docking and molecular dynamics simulations confirm that these compounds interact effectively with key bacterial and fungal proteins. These results suggest that EOLF can be used as a natural source for the development of pharmaceutical, cosmetic and food products. In future, its insecticidal, repellent or corrosive potential could be explored, as well as its use in food preservation, biological control of pathogens and the development of natural formulations with antimicrobial and antioxidant activity.

## Figures and Tables

**Table 1:** Phytochemical composition identified in EOLF.

Chemical Names	Chemical Formulas	Retention Time (min)	RI	Area (%)	CAS Number	Chemical Structure
**p-cymene**	C_10_H_14_	15.56	1024	1.364	99–87–6	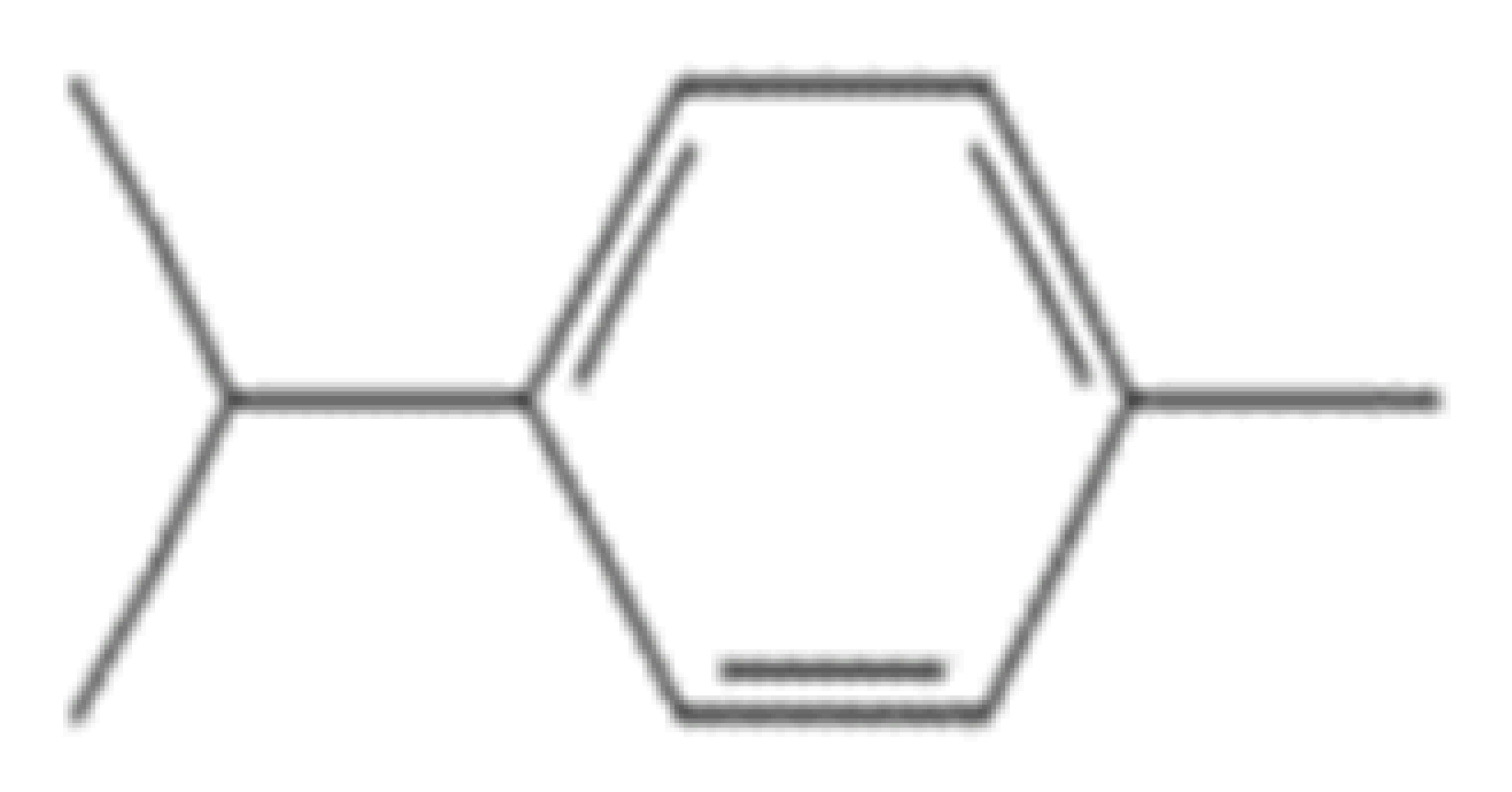
**Eucalyptol**	C_10_H_18_O	15.78	1031	0.125	470–82–6	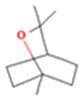
**Terpinene**	C_10_H_16_	16.62	1017	1.423	99–84–3	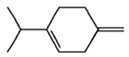
**Cis-sabinene**	C_10_H_16_	18.59	1070	17.063	3387–41–5	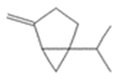
**α-campholenol**	C_10_H_18_O	19.56	1126	6.157	1901–38–8	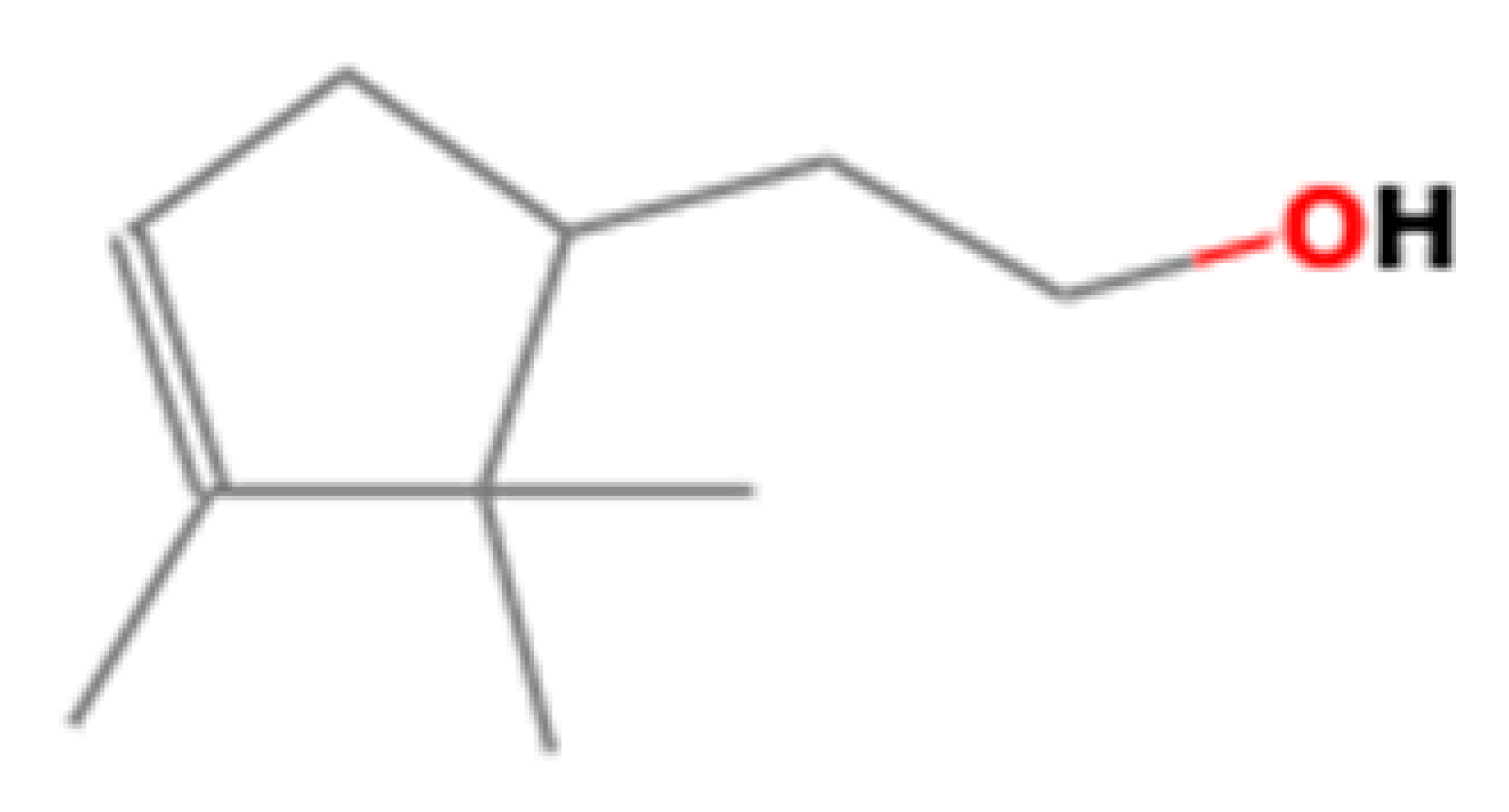
**Isoborneol**	C_10_H_18_O	20.22	1160	4.983	124–76–5	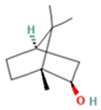
**Borneol**	C_10_H_18_O	20.47	1169	2.897	464–43–7	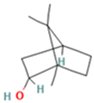
**isopulegone**	C_10_H_16_O	21.34	1237	0.143	29606–79–9	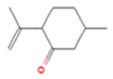
**Myrtenyl acetate**	C_12_H_18_O	23.05	1329	1.684	35670–93–0	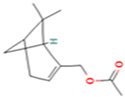
**Carvacrol**	C_10_H_14_O	24.56	1299	35.963	499–75–2	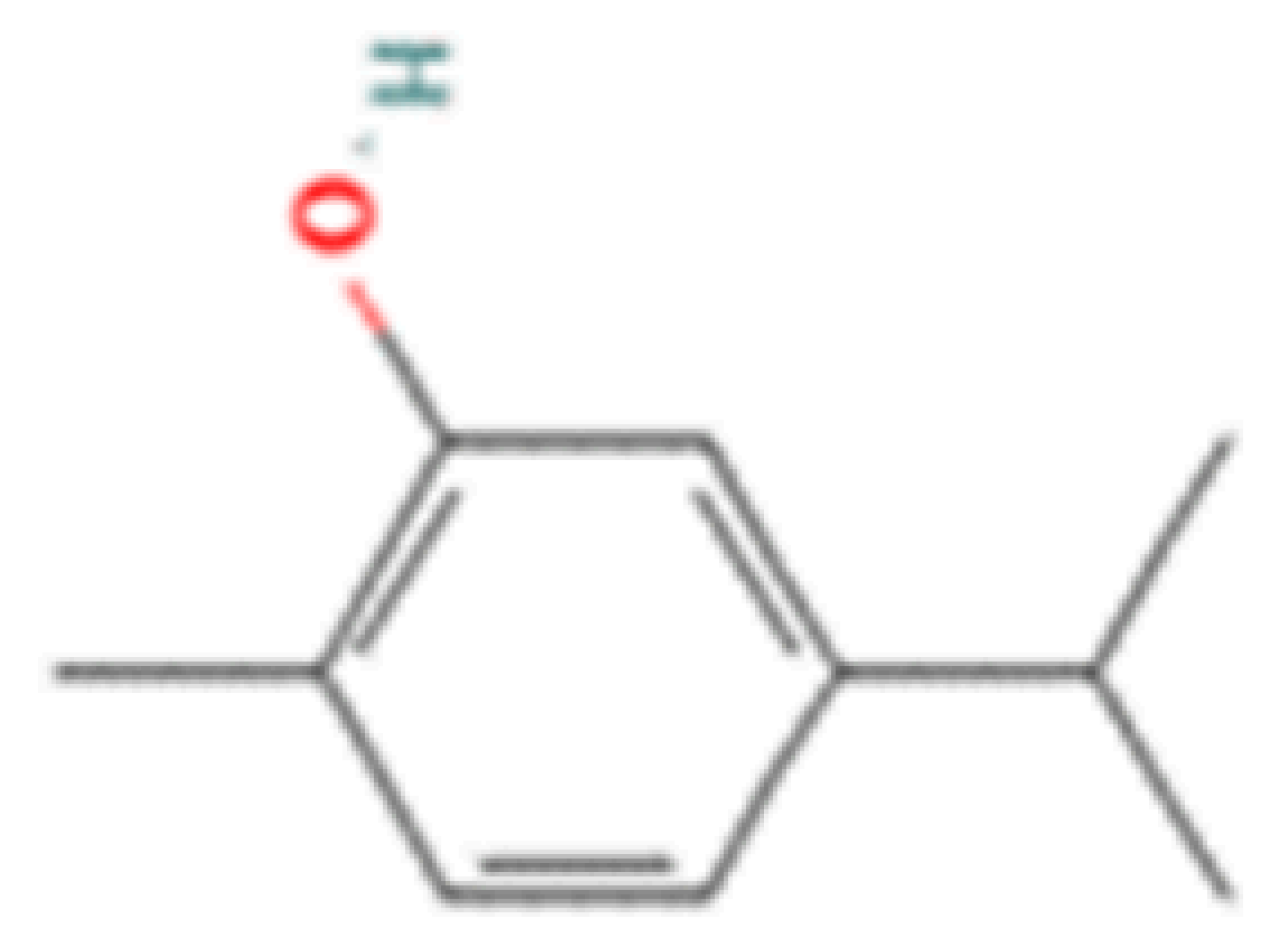
**Caryophyllene**	C_15_H_24_	27.33	1419	15.376	87–44–5	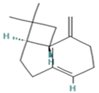
**Valencene**	C_15_H_24_	27.82	1496	0.218	4630–07–3	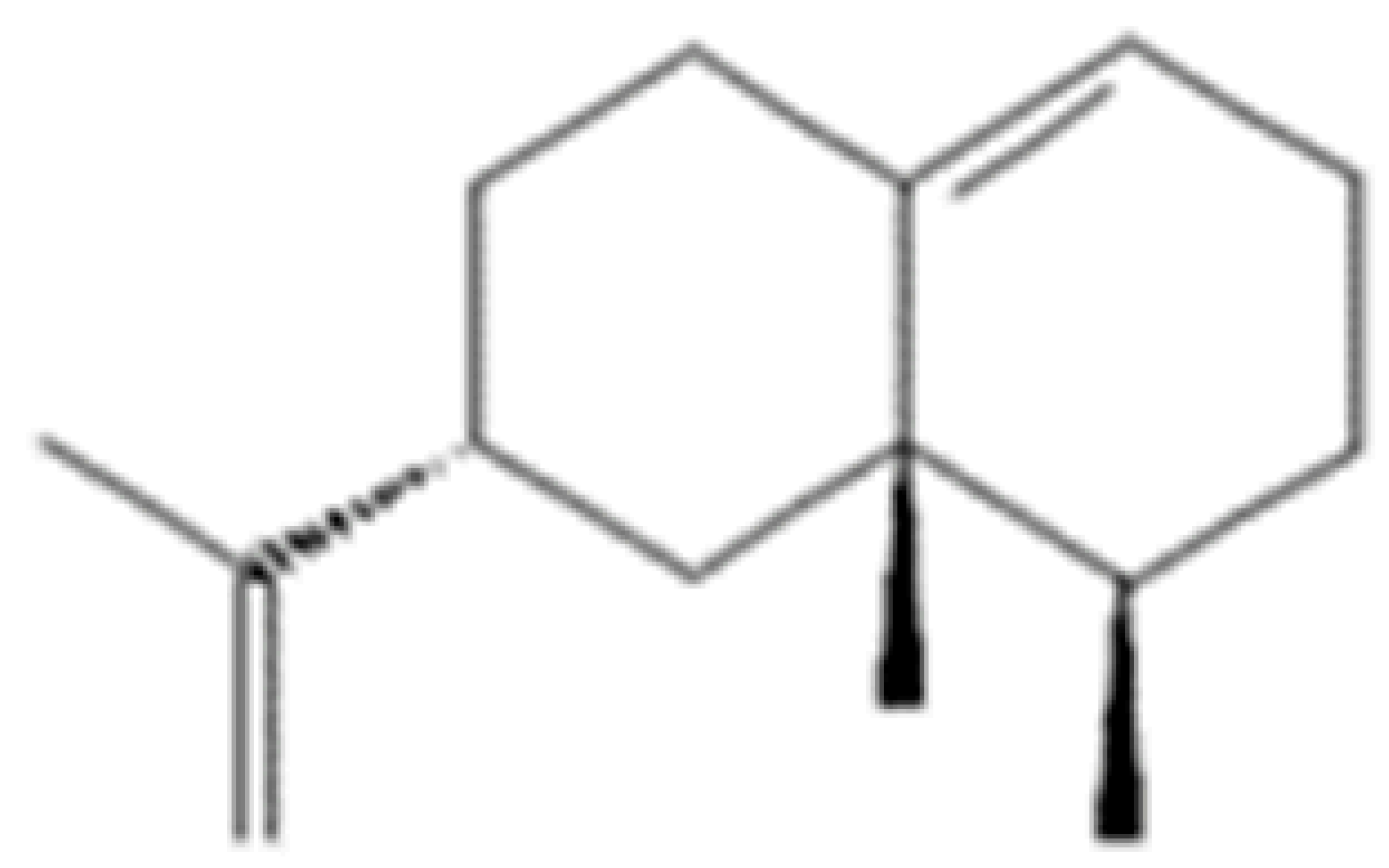
**γ- Muurolene**	C_15_H_24_	29.23	1479	1.637	30021–74–0	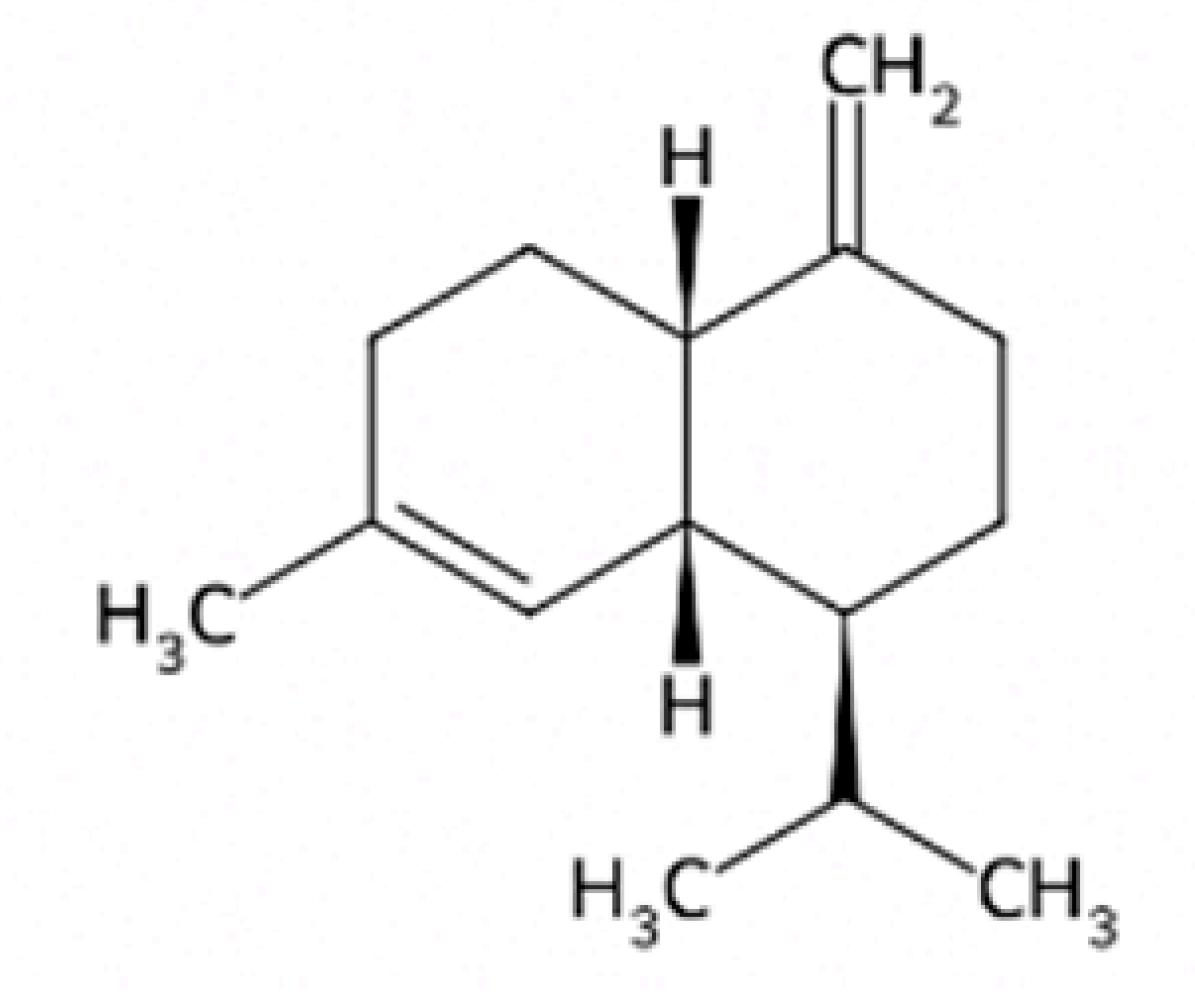
**Caryophyllene oxide**	C_25_H_24_O	31.55	1583	10.921	1139–30–6	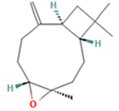
		**Total**		**99.954%**		

**Table 2:** Antimicrobial activity of EOLF in liquid medium determined by MICs.

	EOFL	Stpt	Fluco	Negative Control
***Antibacterial potential in terms of MIC (*** **µg/mL** ***)***				
***S. aureus***	11.03 ± 0.27	12.59 ± 0.71	NT	0.0 ± 0.0
***E. coli***	13.58 ± 1.47	10.44 ± 0.59	NT	0.0 ± 0.0
***K. pneumoniae***	15.68 ± 0.83	0.0 ± 0.0	NT	0.0 ± 0.0
***P. aeruginosa***	16.29 ± 0.79	0.0 ± 0.0	NT	0.0 ± 0.0
***Antifungal potential in terms of MIC (*** **µg/mL** ***)***				
***C. albicans***	10.51 ± 0.47	NT	9.24 ± 0.34	0.0 ± 0.0
***A. niger***	12.88 ± 0.93	NT	10.70 ± 0.77	0.0 ± 0.0
***F. oxysporum***	11.48 ± 0.62	NT	10.97 ± 0.84	0.0 ± 0.0

**EOLF: *Lycium ferocissimum* essential oil; NT: not tested; Fluco: fluconazole; Stpt: streptomycin.**

**Table 3:** Binding scores of the compounds and the reference drug against the antifungal enzymes and anti-bacterial enzymes.

	Cis-Sabinene	α-Campholenol	Isoborneol	Carvacrol	Caryophyllene	Caryophyllene Oxide	Reference Drug
***S. aureus***	−5.6	−5.7	−4.6	−6.0	−6.6	−6.7	−5.6
***E. coli***	−5.6	−5.2	−4.6	−5.9	−5.7	−5.7	−5.8
***K. pneumoniae***	−5.3	−5.7	−5.2	−6.2	−6.4	−6.4	−6.7
***P. aeruginosa***	−6.3	−6.3	−5.2	−6.3	−8.0	−7.2	−6.1
***C. albicans***	−5.5	−5.6	−5.1	−5.6	−6.3	−7.1	−6.7
***A. niger***	−5.4	−5.9	−4.6	−6.3	−6.2	−6.0	−6.4
***F. oxysporum***	−4.9	−4.6	−3.9	−5.2	−5.3	−5.5	−5.2

## Data Availability

Data is contained within the article.

## Supplementary Materials

The following supporting information can be downloaded at: https://www.mdpi.com/article/10.3390/molecules31183224/s1, Table S1: ADMET Predictions for the Proposed Molecules; Figure S1: RMSD trajectories of complexes with compounds (EOLF) on the 100 ns MD simulation; Figure S2: RMSF trajectories of complexes with compounds (EOLF) on the 100 ns MD simulation; Figure S3: Protein–ligand contact of complexes with compounds (EOLF) on the 100 ns MD simulation.

## Abbreviations

The following abbreviations are used in this manuscript:

EOLF	Essential Oil of *L. Ferocissimum*
GC-MS	Gas Chromatography coupled to Mass Spectrometry
DPPH	2,2-diphenyl-1-picrylhydrazyl
FRAP	Ferric Reducing Antioxidant Power
TAC	Total Antioxidant Capacity
MIC	Minimum Inhibitory Concentration
ADMET	Absorption, Distribution, Metabolism, Excretion and Toxicity
MD	Molecular Dynamics
