# Supplementary material for: Antioxidant and Antimicrobial Activities of Essential Oil Extracted from Leaves of African Boxthorn (Lycium ferocissimum): In-Vitro and In-Silico Approaches
Source: Molecules. 2026 Sep 13;31(18):3224. doi: 10.3390/molecules31183224 (PMC13609064; doi:10.3390/molecules31183224)
Supplement: Supplementary file 1 [file molecules-31-03224-s001.zip › molecules-4493435-supplementary.pdf]

Antioxidant and Antimicrobial Activities of Essential Oil Extracted from Leaves of African Boxthorn (*Lycium ferocissimum*): in-vitro and in-silico approaches

Walid El Mjiyad [1], Hind Zejli [2], Meriem Khedraoui [3], Outmane El Omry [4,5], Abderrahim El Yahyaoui [6], Khalid Chebbac [6], Mourad Akdad [7], Salaheddine Chebaibi [1], Mourad A. M. Aboul-Soud [8], John P. Giesy [9,10,11], Mohammed Chalkha [12,*], Dunia A. Al Farraj [13], Samir Chtita [3], Mohammed L'bachir El Kbiach [1], Brahim El Bouzdoudi [1] and Abdelfattah El Moussaoui [1,*]

1. Plant Biotechnology Team, Faculty of Sciences, Abdelmalek Essaadi University, Tetouan 93002, Morocco; walid.elmjiyad@etu.uae.ac.ma (W.E.M), salaheddine.chebaibi@etu.uae.ac.ma (Sd.C.), melkbiach@uae.ac.ma (M.L.E.K.), belbouzdoudi@uae.ac.ma (B.E.B.), a.elmoussaoui@uae.ac.ma (A.E.M)
2. Laboratory of Engineering, Electrochemistry, Modeling and Environment, Faculty of Sciences Dhar El Mahraz, Sidi Mohammed Ben Abdallah University, Fez, Morocco; hind.zejli@usmba.ac.ma (H.Z.)
3. Laboratory of Analytical and Molecular Chemistry, Faculty of Sciences Ben M'Sik, Hassan II University of Casablanca, Casablanca, Morocco; meriemkhedraoui5@gmail.com (M.K.), samirchtita@gmail.com (S.C.)
4. Laboratory of Biotechnology and Environment, Agri-Food and Health, Faculty of Sciences Dhar El Mahraz, Sidi Mohammed Ben Abdellah University, P.O. Box 1796 (Atlas), 30000 Fez, Morocco; outmane.elomry@usmba.ac.ma (O.E.O.)
5. Ecology Unit, Department of Plant Protection and Environment, National School of Agriculture of Meknes, BP S 40, Meknes, Morocco; outmane.elomry@usmba.ac.ma (O.E.O.)
6. Laboratory of Biotechnology, Conservation and Valorisation of Natural Resources, Faculty of Sciences Dhar El Mahraz, Sidi Mohammed Ben Abdallah University, Fez, Morocco; abderrahim.elyahyaoui@usmba.ac.ma (A.E.Y), khalid.chebbac@usmba.ac.ma (K.C.)
7. Department of Biology, Faculty of Sciences and Techniques Errachidia, Moulay Ismail University of Meknes, BP 509, Boutalamine, 52000. Errachidia, Morocco; akdad.mourad@gmail.com (M.A.)
8. Center of Excellence in Biotechnology Research, College of Applied Medical Sciences, King Saud University, Riyadh, Saudi Arabia; maboulsoud@ksu.edu.sa (M.A.M.A.)
9. School of Earth and Environmental Sciences, Baylor University, Waco, TX 76798, USA; jgiesy@aol.com (J.P.G.)
10. Department of Veterinary Biomedical Sciences and Toxicology Centre, University of Saskatchewan, Saskatoon S7N 5B3, Canada.
11. Department of Integrative Biology and Center for Integrative Toxicology, Michigan State University, East Lansing, MI 48823, USA.; e-mail@e-mail.com
12. Laboratory of Materials Engineering for the Environment and Natural Resources, Faculty of Sciences and Techniques, University of Moulay Ismail of Meknes, B.P 509, Boutalamine, 52000, Errachidia, Morocco; mohammed.chalkha1@usmba.ac.ma (M.C.)
13. Department of Botany and Microbiology, College of Science, King Saud University, P.O. Box 2455, Riyadh 11451, Saudi Arabia; dfarraj@ksu.edu.sa (D.A.A.)

* Correspondence: mohammed.chalkha1@usmba.ac.ma (M.C.); a.elmoussaoui@uae.ac.ma (A.E.M)

Table S1. ADMET Predictions for the Proposed Molecules

	Molecular weight (g/mol) weight (g/mol)	LOGP	HBA	HBD	Caco2	HIA %	VDss	BBB	CYP2D6 inhibitior	CYP3A4 inhibitior	Total Clearance	AMES toxicity	LD_{50} (mol/Kg)	Hepatotoxicity
Cis-sabinene	136.238	2.9	0	0	1.397	95.135	0.566	0.837	No	No	0.071	No	1.528	No
α-campholenol	154.253	2.4	1	1	1.493	94.785	0.237	0.599	No	No	0.257	No	1.802	No
Isoborneol	154.253	2.2	1	1	1.209	96.275	0.236	0.626	No	No	1.035	No	2.016	No
Carvacrol	150.221	2.8	1	1	1.068	93.712	0.351	0.381	No	No	0.243	No	1.906	No
Caryophyllene	204.357	4.7	0	0	1.417	95.322	0.659	0.727	No	No	1.088	No	1.604	No
Caryophyllene oxide	220.356	3.9	1	0	1.476	95.458	0.588	0.644	No	No	0.905	No	1.534	No

HBA: Hydrogen bond acceptor, HBD: Hydrogen bond, HIA%: Intestinal absorption (human), VDss (Human): The steady state volume of distribution donor, BBB: Blood-Brain Barrier.

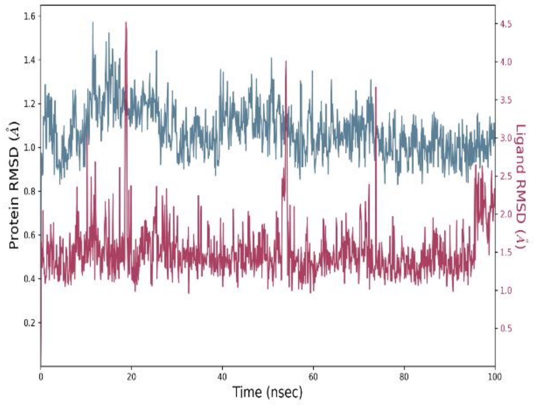
Protein RMSD (Å)
Ligand RMSD (Å)
Time (nsec)
0.2 0.4 0.6 0.8 1.0 1.2 1.4 1.6
0.5 1.0 1.5 2.0 2.5 3.0 3.5 4.0 4.5
0 20 40 60 80 100

A

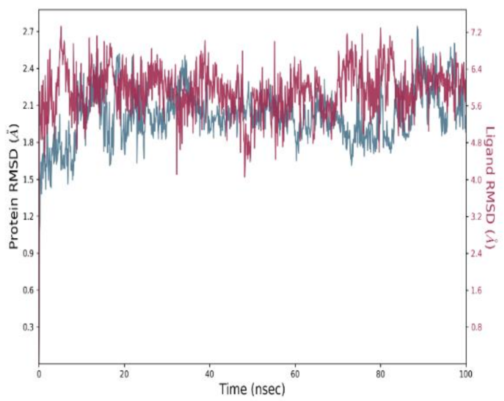
Protein RMSD (Å)
Ligand RMSD (Å)
Time (nsec)
0.3 0.6 0.9 1.2 1.5 1.8 2.1 2.4 2.7
0.8 1.6 2.4 3.2 4.0 4.8 5.6 6.4 7.2
0 20 40 60 80 100

B

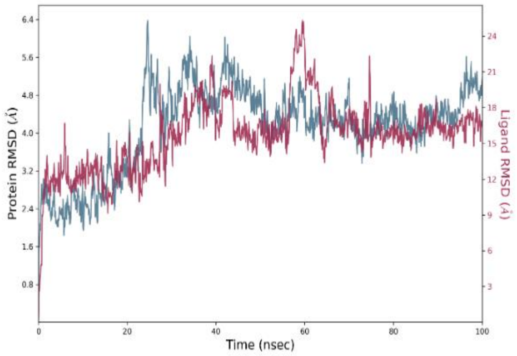
Protein RMSD (Å)
Ligand RMSD (Å)
Time (nsec)
0.8 1.6 2.4 3.2 4.0 4.8 5.6 6.4
3 6 9 12 15 18 21 24
0 20 40 60 80 100

C

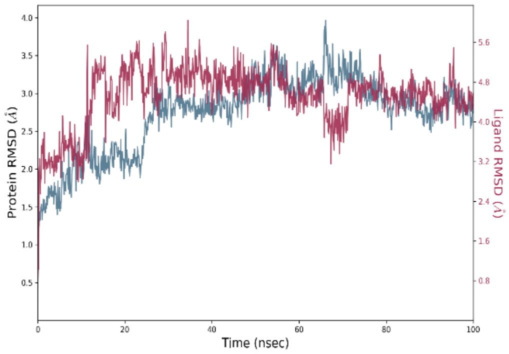
Protein RMSD (Å)
Ligand RMSD (Å)
Time (nsec)
0.5 1.0 1.5 2.0 2.5 3.0 3.5 4.0
0.8 1.6 2.4 3.2 4.0 4.8 5.6
0 20 40 60 80 100

D

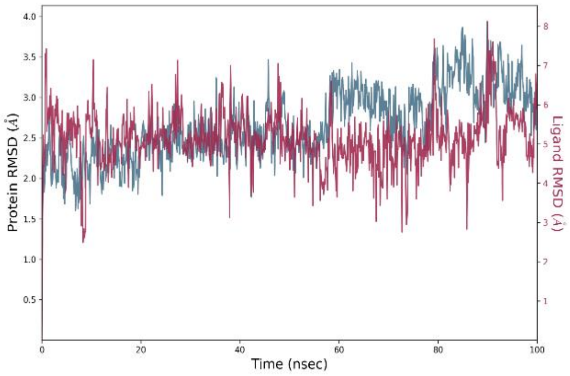
Protein RMSD (Å)
Ligand RMSD (Å)
Time (nsec)
0.5 1.0 1.5 2.0 2.5 3.0 3.5 4.0
1 2 3 4 5 6 7 8
0 20 40 60 80 100

E

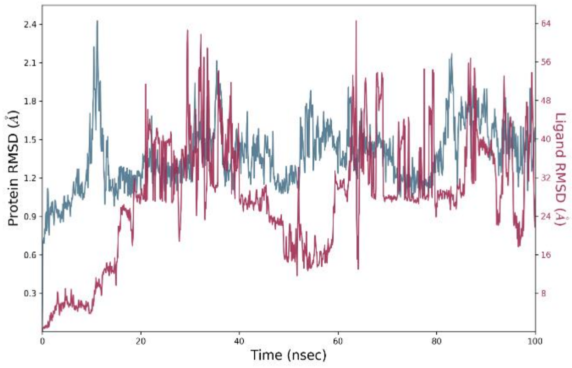
Protein RMSD (Å)
Ligand RMSD (Å)
Time (nsec)
0.3 0.6 0.9 1.2 1.5 1.8 2.1 2.4
8 16 24 32 40 48 56 64
0 20 40 60 80 100

F

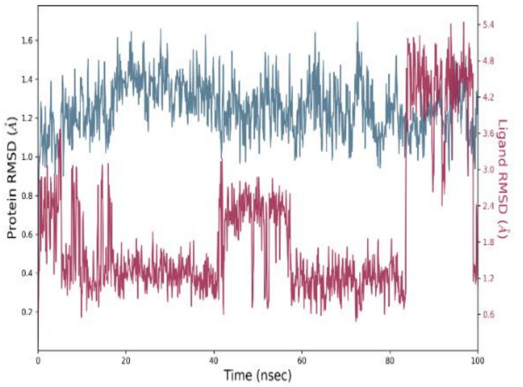

G

Figure S1. RMSD trajectories of complexes with compounds (EOLF) on the 100 ns MD simulation. (A) 3G7B-caryophyllene oxide, (B) 1KZN-α-campholenol, (C) 8JGW-caryophyllene oxide, (D) 6Z5K-caryophyllene, (E) 1EAG-caryophyllene oxide, (F) 7BLY-carvacrol, (G) 8EBB-caryophyllene oxide.

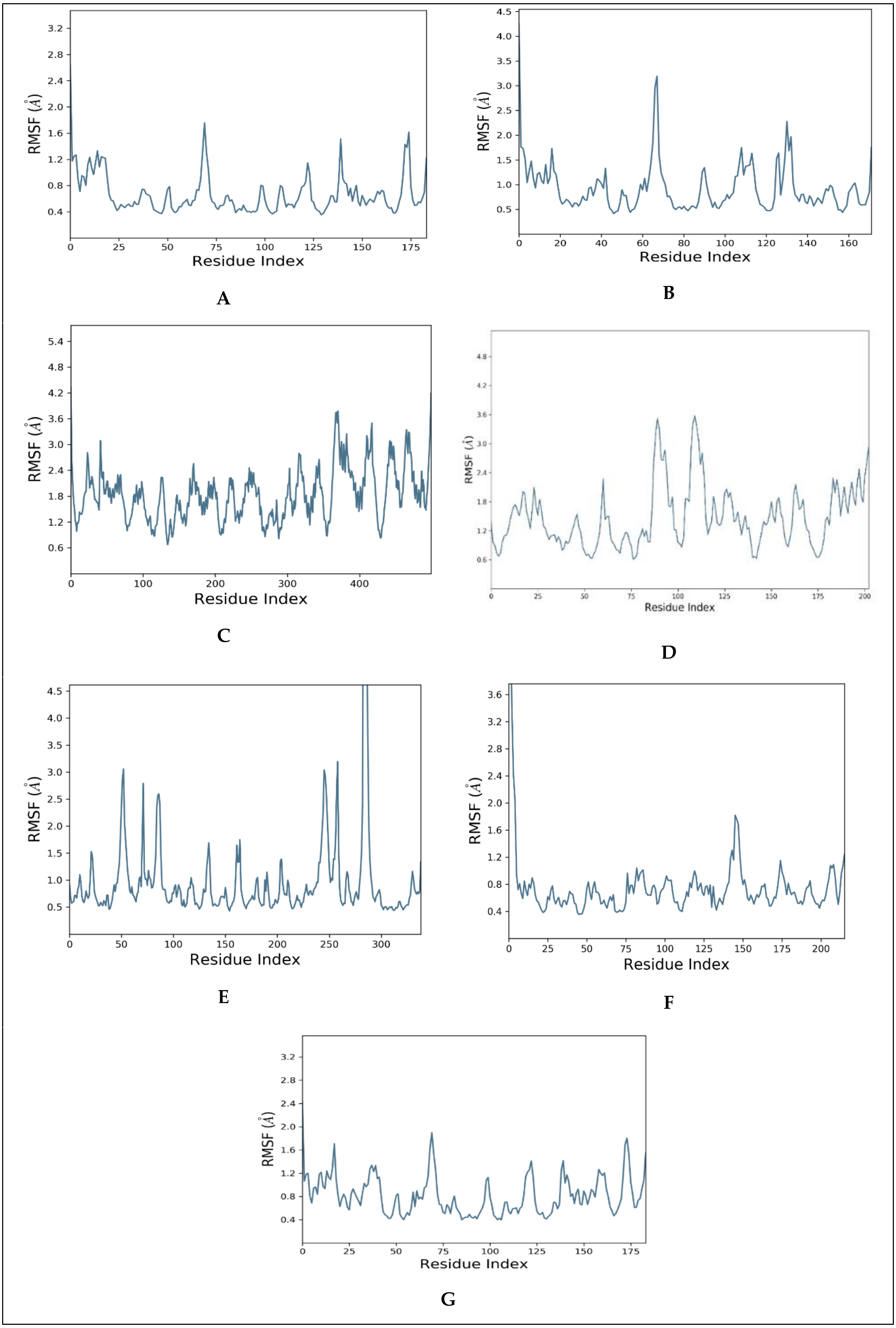
RMSF (Å)
Residue Index
3.2
2.8
2.4
2.0
1.6
1.2
0.8
0.4
0
25
50
75
100
125
150
175
A
4.5
4.0
3.5
3.0
2.5
2.0
1.5
1.0
0.5
0
20
40
60
80
100
120
140
160
B
5.4
4.8
4.2
3.6
3.0
2.4
1.8
1.2
0.6
0
100
200
300
400
C
4.8
4.2
3.6
3.0
2.4
1.8
1.2
0.6
0
25
50
75
100
125
150
175
200
D
4.5
4.0
3.5
3.0
2.5
2.0
1.5
1.0
0.5
0
50
100
150
200
250
300
E
3.6
3.2
2.8
2.4
2.0
1.6
1.2
0.8
0.4
0
25
50
75
100
125
150
175
200
F
3.2
2.8
2.4
2.0
1.6
1.2
0.8
0.4
0
25
50
75
100
125
150
175
G

Figure S2. RMSF trajectories of complexes with compounds (EOLF) on the 100 ns MD simulation. (A) 3G7B-caryophyllene oxide, (B) 1KZN-α-campholenol, (C) 8JGW-caryophyllene oxide, (D) 6Z5K-caryophyllene, (E) 1EAG-caryophyllene oxide, (F) 7BLY-carvacrol, (G) 8EBB-caryophyllene oxide.

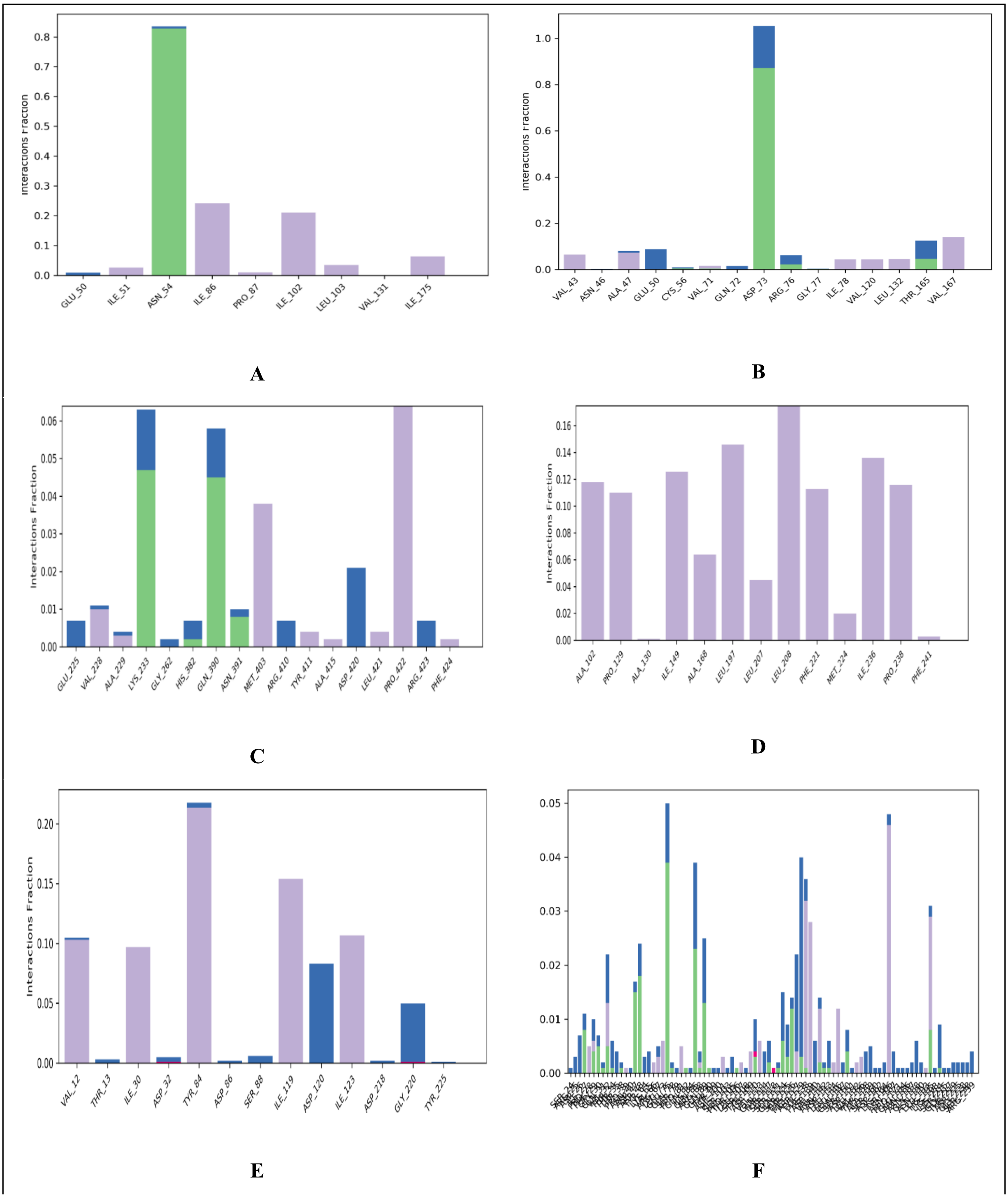
Interactions Fraction
GLU_50
ILE_51
ASN_54
ILE_86
PRO_87
ILE_102
LEU_103
VAL_131
ILE_175
A
VAL_43
ASN_46
ALA_47
GLU_50
CYS_56
VAL_71
GLN_72
ASP_73
ARG_76
GLY_77
ILE_78
VAL_120
LEU_132
THR_165
VAL_167
B
GLU_225
VAL_228
ALA_229
LYS_233
GLY_262
HIS_382
GLN_390
ASN_391
MET_403
ARG_410
TYR_411
ALA_415
ASP_420
LEU_421
PRO_422
ARG_423
PHE_424
C
ALA_102
PRO_129
ALA_130
ILE_149
ALA_168
LEU_197
LEU_207
LEU_208
PHE_221
MET_224
ILE_236
PRO_238
PHE_241
D
VAL_12
THR_13
ILE_30
ASP_32
TYR_84
ASP_86
SER_88
ILE_119
ASP_120
ILE_123
ASP_218
GLY_220
TYR_225
E
F

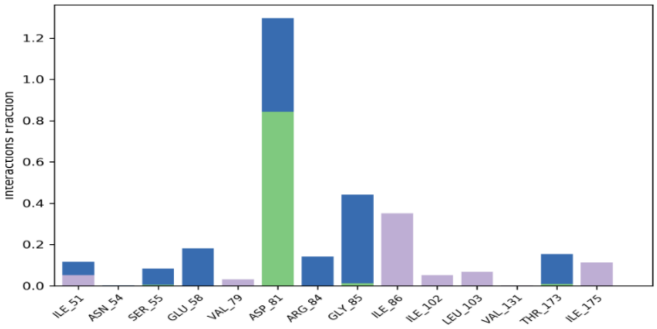

G

Figure S3. Protein–ligand contact of complexes with compounds (EOLF) on the 100 ns MD simulation. (A) 3G7B -caryophyllene oxide, (B) 1KZN-α-campholenol, (C) 8JGW-caryophyllene oxide, (D) 6Z5K-caryophyllene, (E) 1EAG-caryophyllene oxide, (F) 7BLY-carvacrol, (G) 8EBB-caryophyllene oxide.
